# Supplementary figures and images for: Expression of intron-containing HIV-1 RNA induces NLRP1 inflammasome activation in myeloid cells
Source: PLoS Biol. 2025 Sep 8;23(9):e3003320. doi: 10.1371/journal.pbio.3003320 (PMC12416851; doi:10.1371/journal.pbio.3003320)

S1 Figure

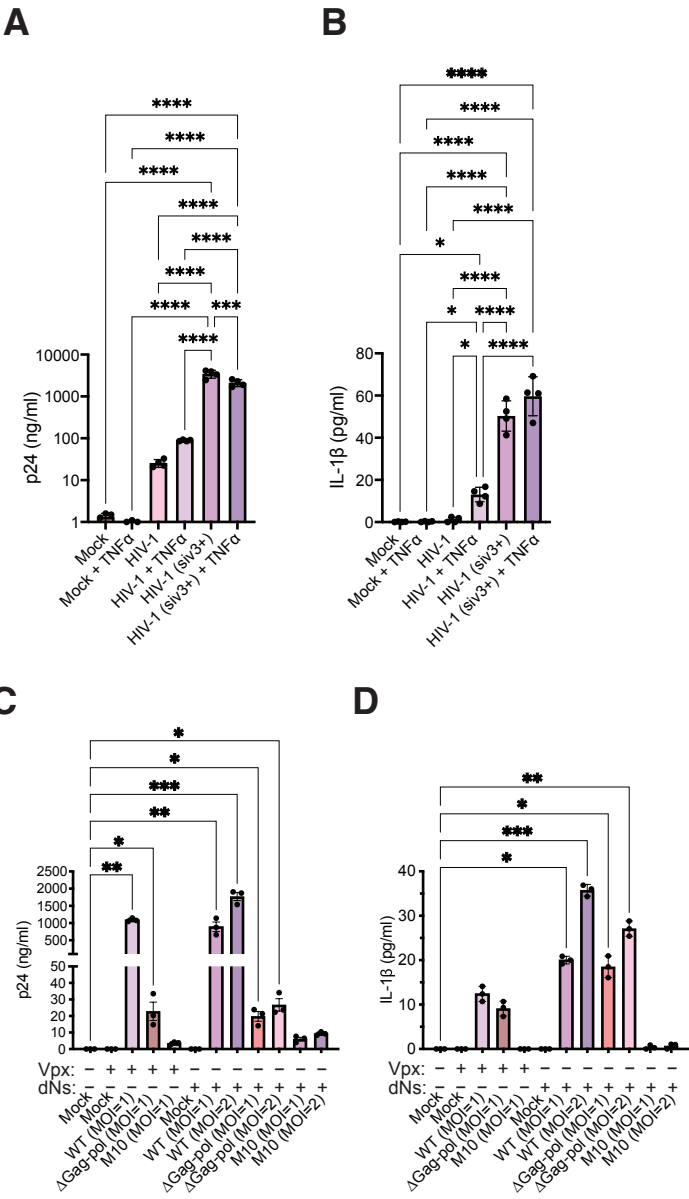

Supplement: S1 Fig — Quantitation of p24Gag release (A and C) and IL-1β secretion (B and D) by ELISA in culture supernatants from MDMs that were either stimulated with or without TNFα (10 ng/ml), pre-treated with either Vpx-VLPs or deoxynucleosides (dNs) before infection with Lai∆envGFP/G (WT, ∆Gag-pol, or M10), was quantified at d3pi. The means ± SEM of 3 independent experiments are shown with cells derived from 3 independent donors. Statistical significance was determined by one-way ANOVA followed by Tukey’s multiple comparisons test (A and B), or the Kruskal–Wallis test, comparing uninfected (mock) to WT or mutant HIV-infected MDMs in both Vpx and dNs-treated conditions. P-values: *** < 0.001; ** < 0.01; * < 0.05; no symbol: not significant (p ≥ 0.05). The data underlying this figure can be found in S1 Supplementary Data. (PDF) [file pbio.3003320.s001.pdf]

S2 Figure

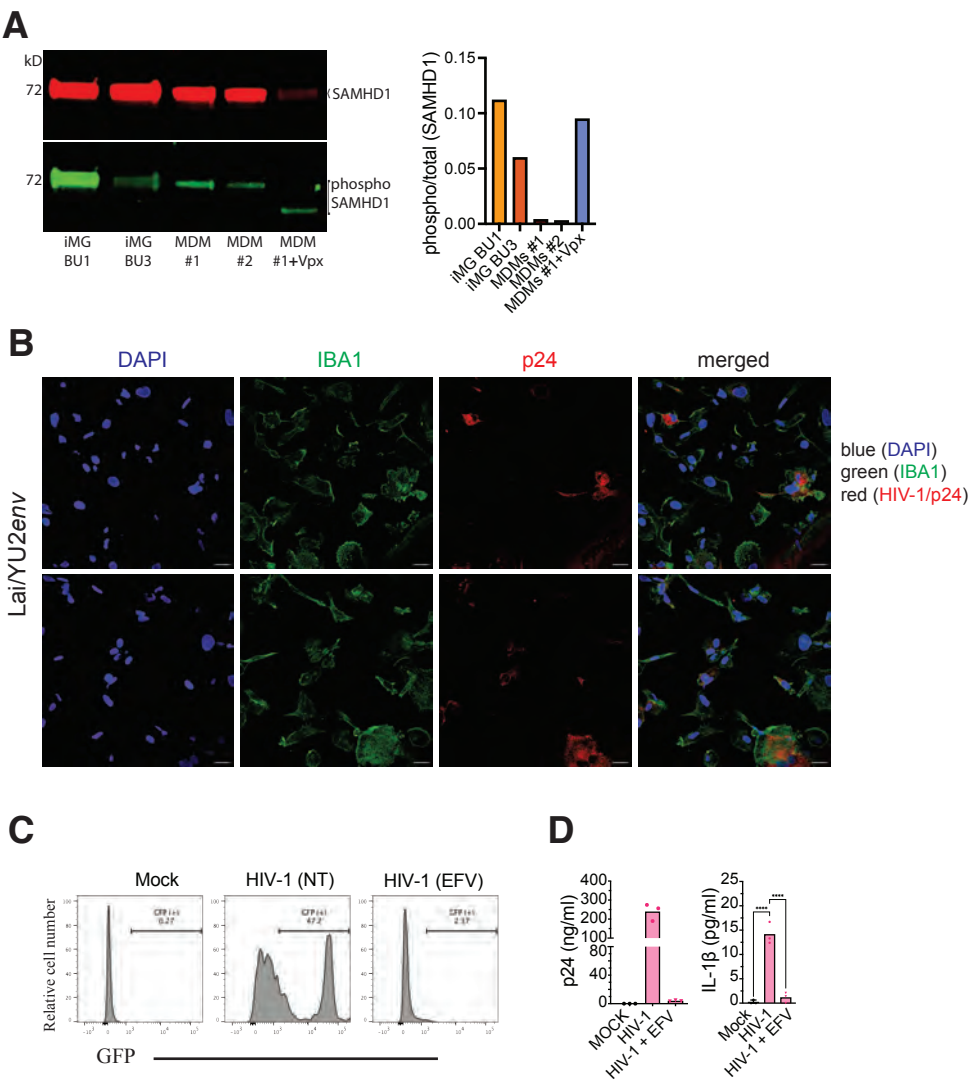

Supplement: S2 Fig — (A) Immunoblotting (left panel) and quantitation (right panel) for unphosphorylated and phosphorylated forms of SAMHD1 in iMGs from 2 iPSC lines (BU1 and BU3) and MDMs from two different donors ± Vpx-VLPs. (B) Representative immunofluorescence images of iMGs infected with replication-competent Lai/YU-2env (MOI = 1) and analyzed by confocal microscopy. Cells were stained for intracellular p24Gag, IBA1, and DAPI. Scale bar represents 10 μm. (C) Infection of iMGs (BU3) with Lai∆envGFP/G (MOI 1) in the absence (no treatment, NT) or presence of EFV (1μM) for 3 days, analyzed by flow cytometry for GFP expression. (D) ELISA for p24Gag (left panel) and IL-1β (right panel) expression in supernatants from Lai∆envGFP/G-infected iMGs. The data is reported as mean ± SEM from 3 independent experiments from iMGs derived from 2 donor lines. Statistical significance was determined by one-way ANOVA followed by Dunnett’s post-test compared to HIV-infected iMGs (D). P-values: **** < 0.0001; no symbol: not significant (p ≥ 0.05). The data underlying this figure can be found in S2 Supplementary Data. (PDF) [file pbio.3003320.s002.pdf]

S3 Figure

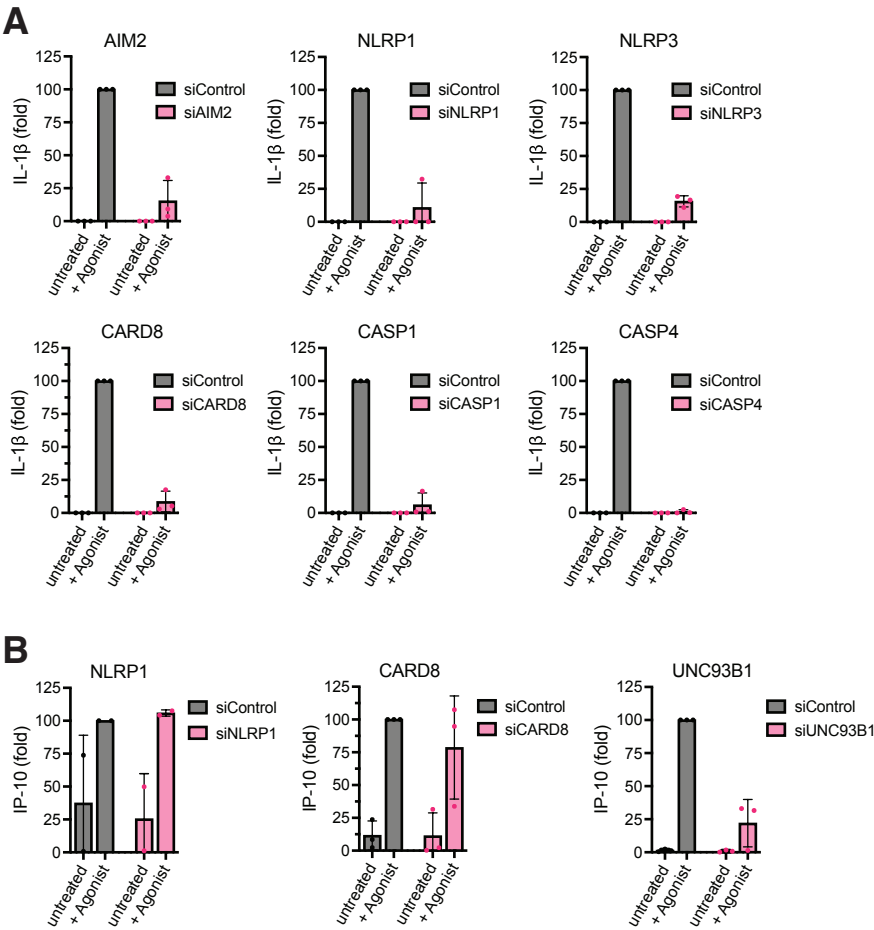

Supplement: S3 Fig — (A and B) Transient knockdown of AIM2, NLRP1, NLRP3, CARD8, Caspase-1, Caspase-4, and UNC93B1 by siRNA transfection in primary MDMs from 3 donors, followed by stimulation with respective agonists for each target (described below). Culture media was harvested at the time points post-stimulation described below. Quantification of IL-1β released in the culture supernatants determined by ELISA (A). ISG induction post-stimulation was determined by quantifying secreted IP-10 by ELISA (B). Stimulations post-siRNA knockdown of MDMs: AIM2, cells were treated with ultra-pure LPS (100 ng/mL) for 2 h, followed by transfection with linearized DNA (1 μg/mL) for 4 h; NLRP1 and CARD8, cells were primed with Pam3CSK4 (0.5 μg/mL) for 4 h, followed by stimulation with VbP (10 μM) for 24 h; NLRP3 and Caspase-1, cells were primed with ATP (5 mM) for 6 h, followed by activation with nigericin (10 μM) for 60 min; Caspase-4, cells were transfected with ultra-pure LPS (5 μg/ml) for 6 h; UNC93B1, cells were treated with Resiquimod (5 μg/ml) for 24 h. For each knockdown and stimulation, IL-1β or IP-10 secretion from siControl-transfected MDMs was set at 100, and the fold decrease upon inflammasome component knockdown was reported. The means ± SEM are shown, and each symbol represents an independent donor. The data underlying this figure can be found in S3 Supplementary Data. (PDF) [file pbio.3003320.s003.pdf]

S4 Figure

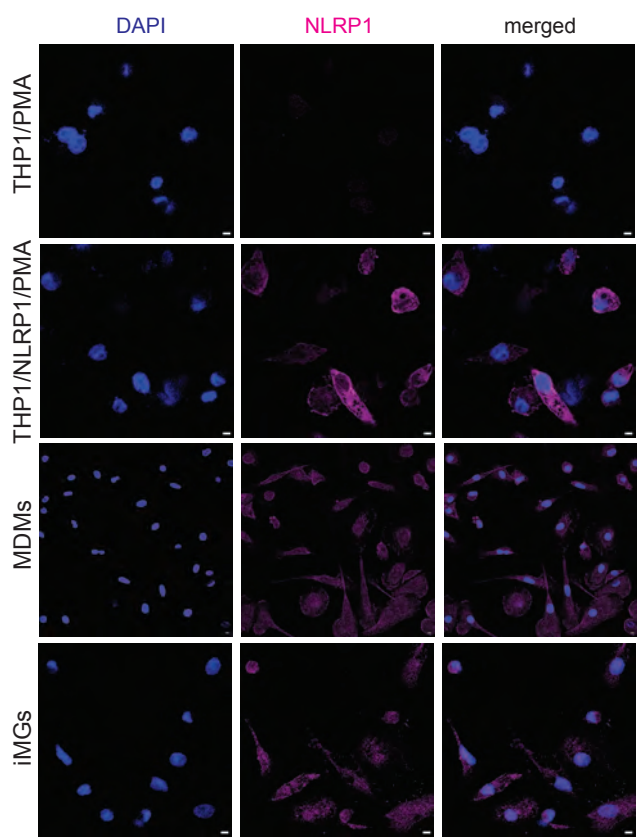

Supplement: S4 Fig — Representative immunofluorescence images of NLRP1 expression in THP-1/PMA macrophages, THP1/PMA/NLRP1 macrophages, MDMs, and iMGs. Scale bar represents 5 μm. (PDF) [file pbio.3003320.s004.pdf]

# S5 Figure

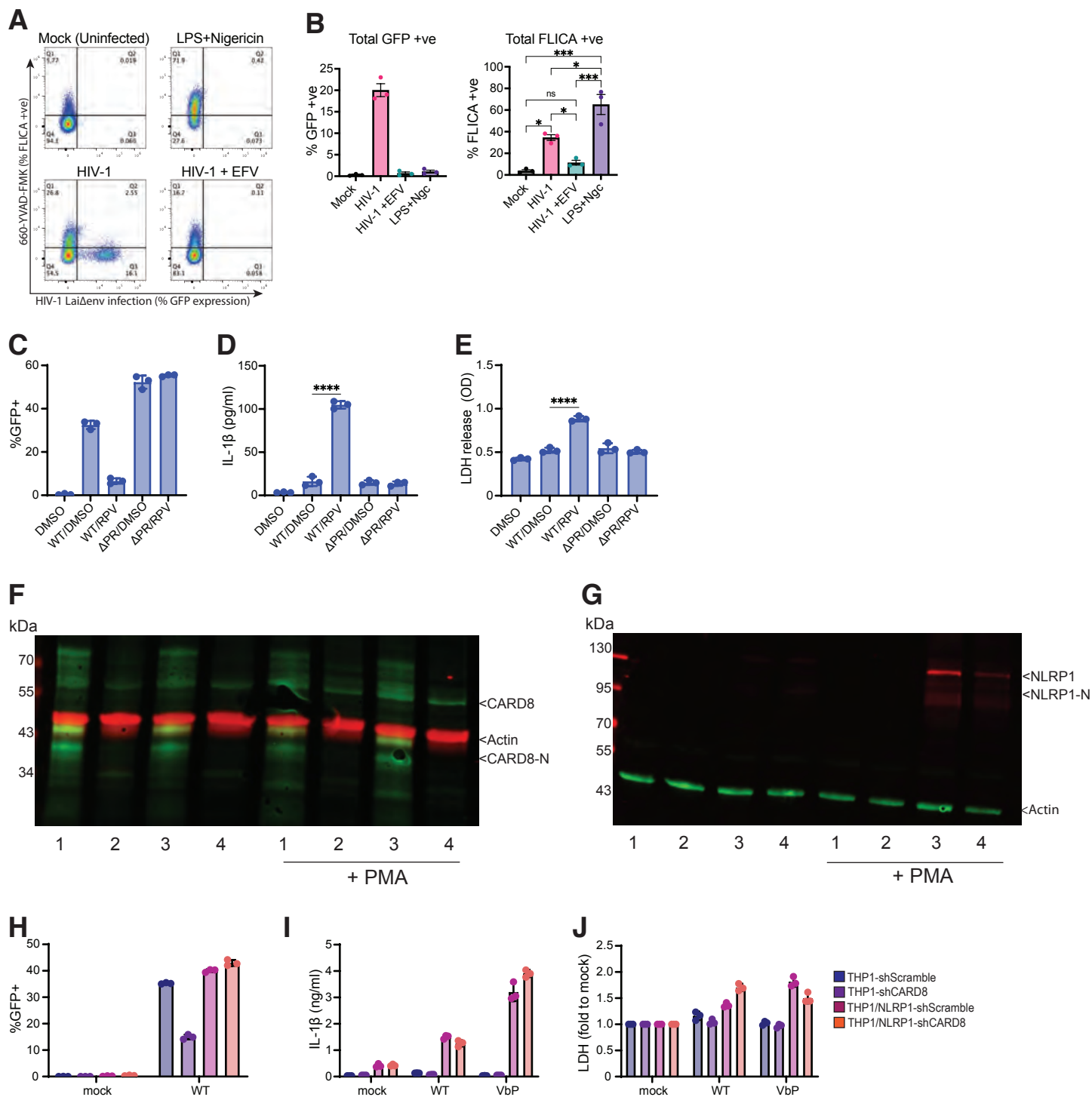

Supplement: S5 Fig — (A, B) PMA-differentiated THP-1/NLRP1 cells were either treated with virus-free media (Mock), infected with Lai∆envGFP/G WT (MOI = 1) in the absence (DMSO) or presence of RT inhibitor, Efavirenz (EFV) for 3 days, or transfected with lipopolysaccharide (LPS, 1 μg) for 24 h, followed by treatment with Nigericin (Ngc, 20 μM) for 30 min. (A) Representative flow cytometry profiles of THP1/NLRP1 cells showing expression of GFP (x-axis) and caspase-1 activation determined by YVAD-660 expression (y-axis). (B) Quantification of % GFP expression (left panel), and % caspase-1 activation (right panel). (C–E) PMA-differentiated THP-1/parental macrophages were treated with 10 ng/mL TNFα and co-infected with wildtype (WT) or protease-inactive mutant (ΔPR) single-cycle HIV-1 and SIV3+/Vpx VLPs for 48h before medium change and treatment with 5 µM RPV or DMSO (control). Graphs show (C) GFP expression, (D) IL-1β secretion, and (E) LDH release after 24h drug treatment. (F, G) western blot showing CARD8 expression (F) or NLRP1 expression (G) in (1) parental THP-1 cells with knockdown (KD) control (shScramble), (2) parental THP-1 cells with CARD8 KD (shCARD8), (3) THP-1/NLRP1 cells without KD (shScramble), or (4) THP-1/NLRP1 cells with CARD8 KD (shCARD8), with or without differentiation with PMA (48 h). (H–J) The 4 lines of THP-1/PMA macrophages shown in (F) and (G) were treated with 10 ng/mL TNFα and infected with Lai∆envGFP/G (MOI = 1) or treated with VbP (100 μM). Graphs show (H) GFP expression, (I) IL-1β secretion, and (J) normalized LDH release 72h post-infection (or 24h post-VbP treatment). Statistical significance was determined by one-way ANOVA followed by Dunnett’s post-test compared to DMSO-treated uninfected control (mock) (D, E). P-values: **** < 0.0001; no symbol: not significant (p ≥ 0.05). The data underlying this figure can be found in S4 Supplementary Data. (PDF) [file pbio.3003320.s005.pdf]

S6 Figure

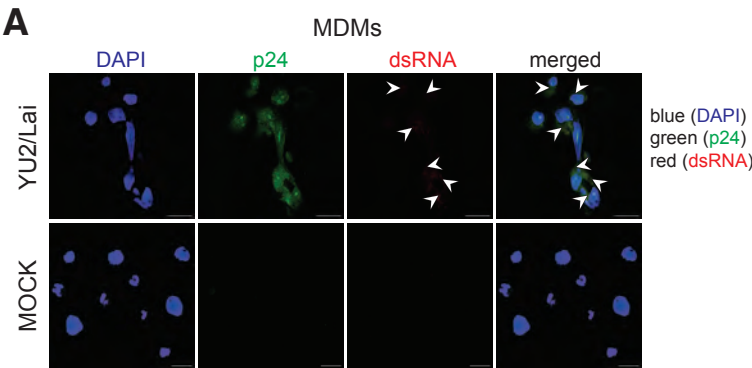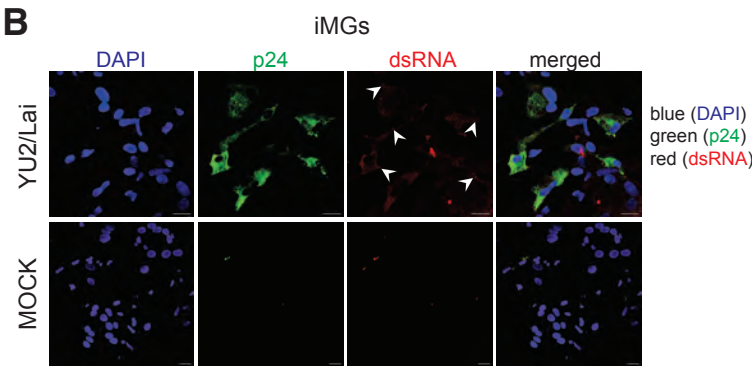

Supplement: S6 Fig — (A, B) Representative immunofluorescence images of MDMs (A) or iMGs (B) infected with Lai/YU-2env (MOI = 1), and stained for intracellular p24Gag, dsRNA, and DAPI at 3 dpi. The white arrowheads indicate dsRNA puncta. Scale bar represents 20 μm. (PDF) [file pbio.3003320.s006.pdf]

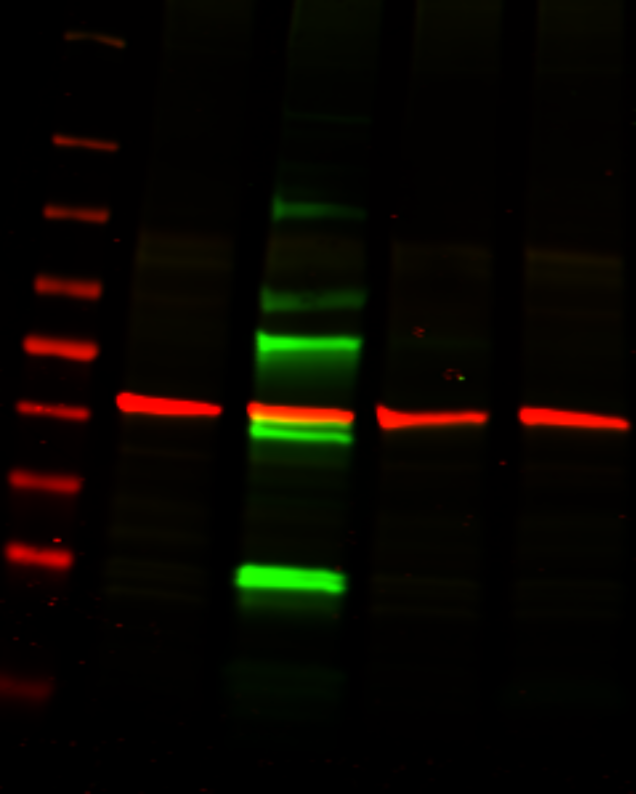

Supplement: S2 Data — (ZIP) [file pbio.3003320.s010.zip › S2_Data/S2_Data_A_HIV-1-Constructs.pdf]

BU1

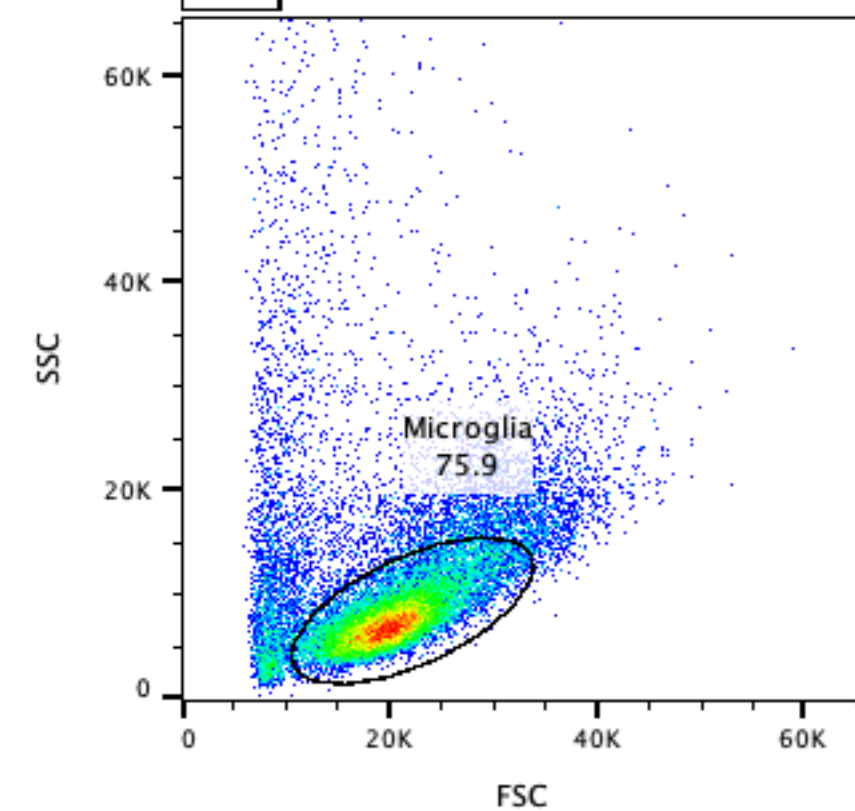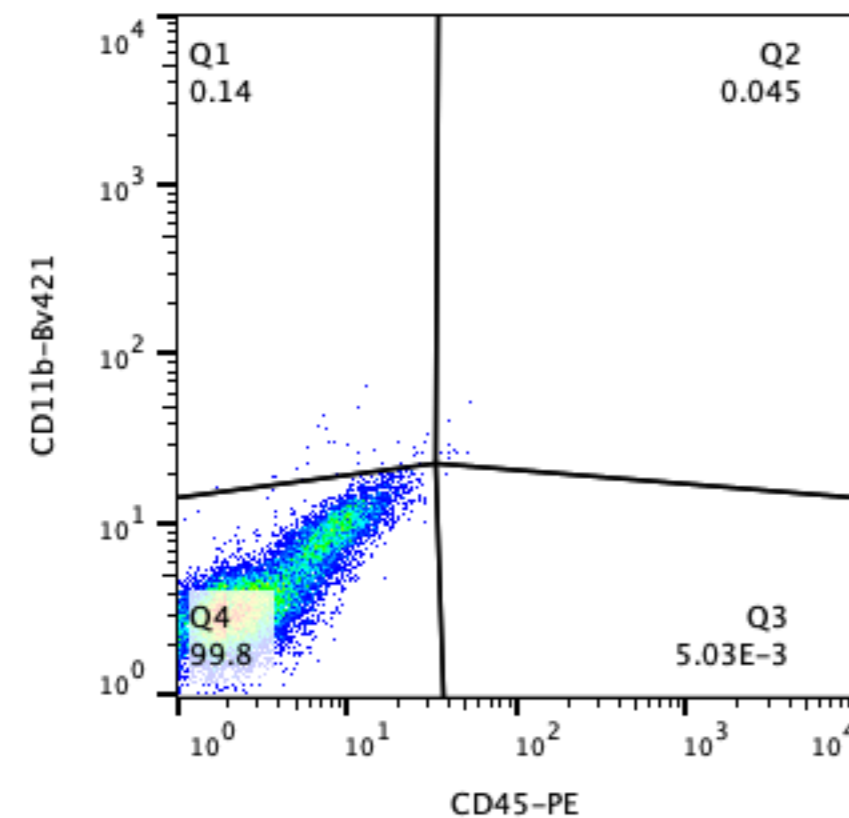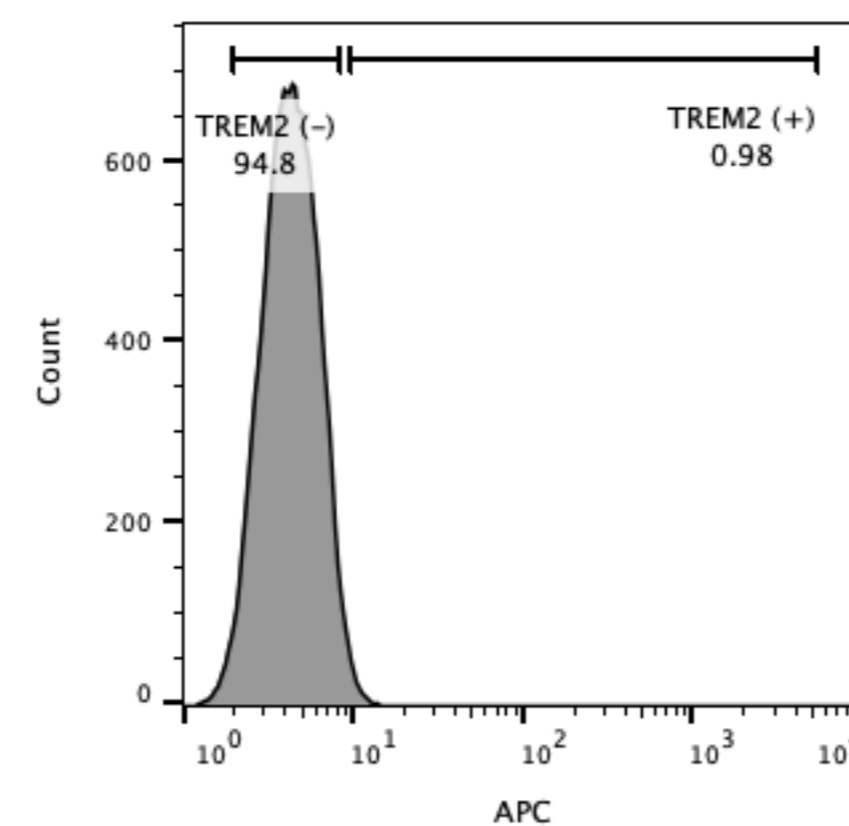

BU3

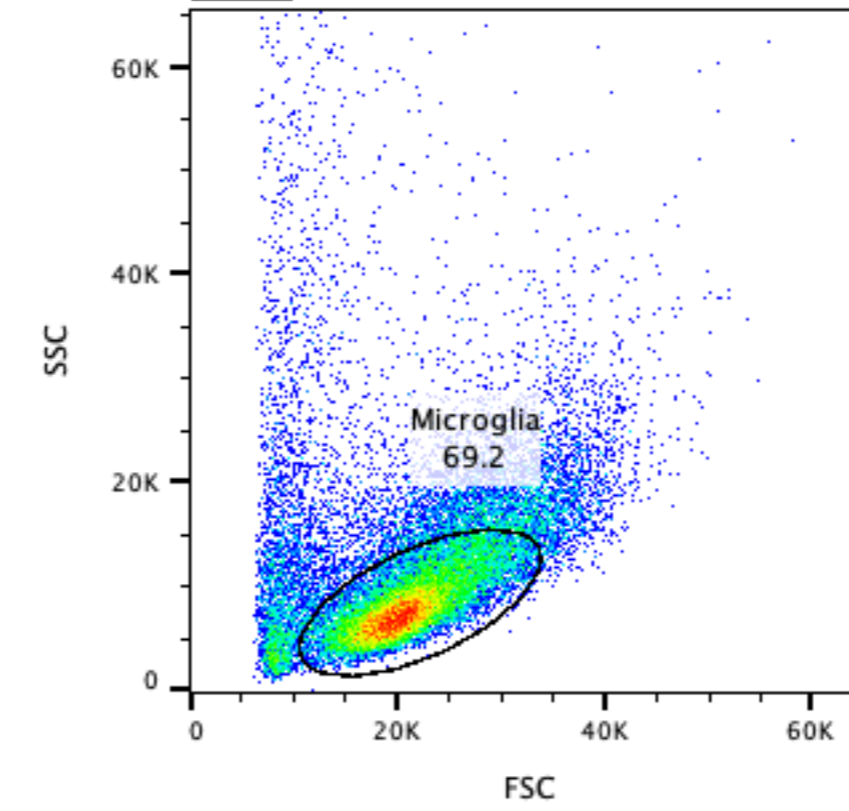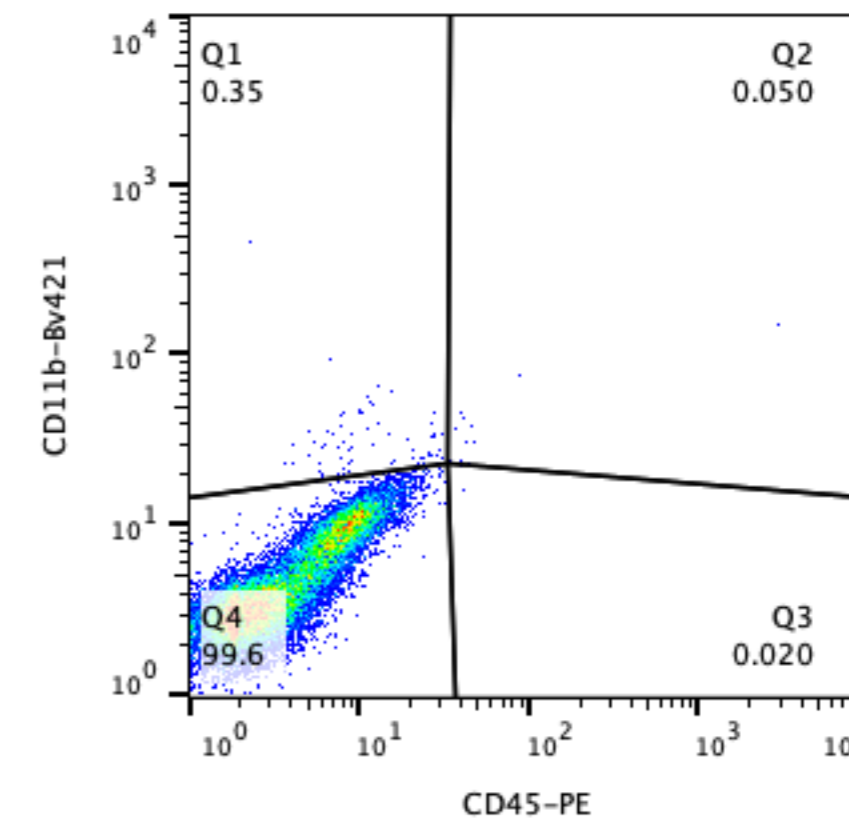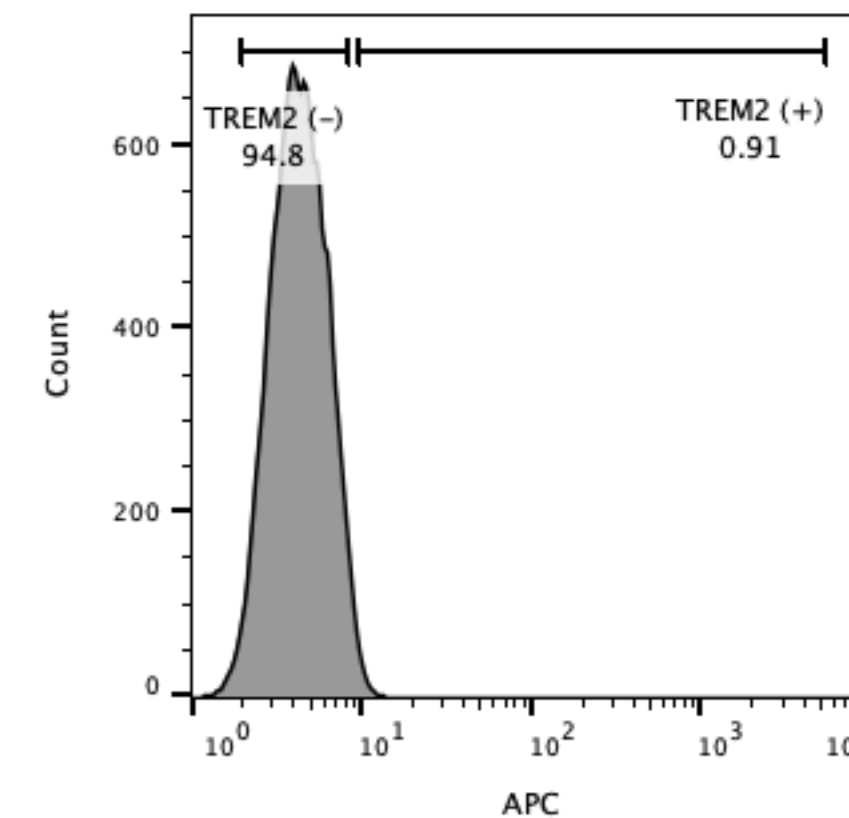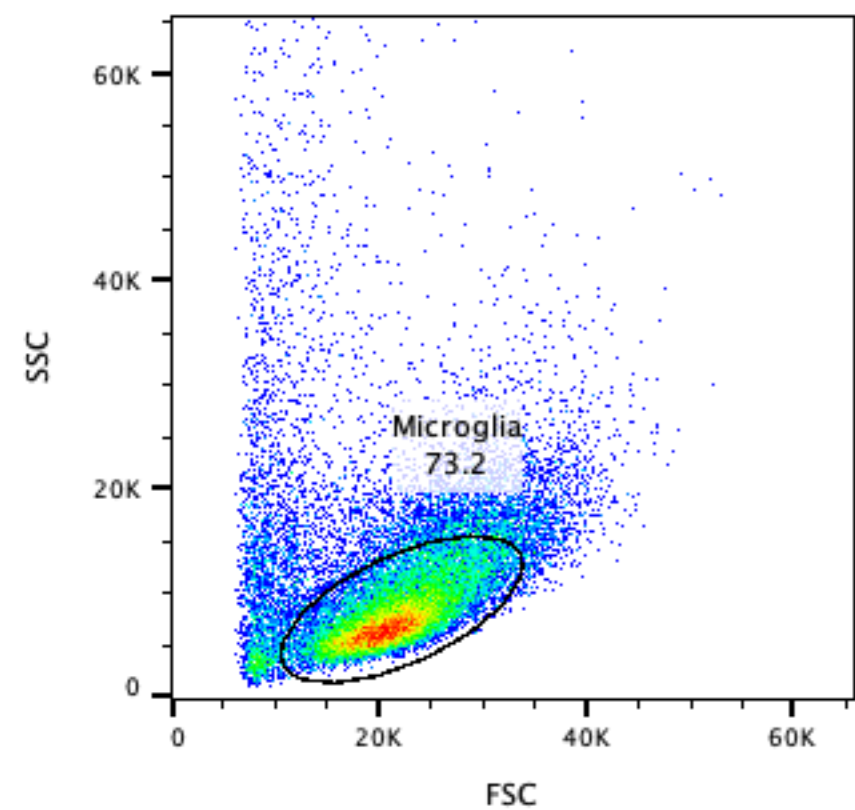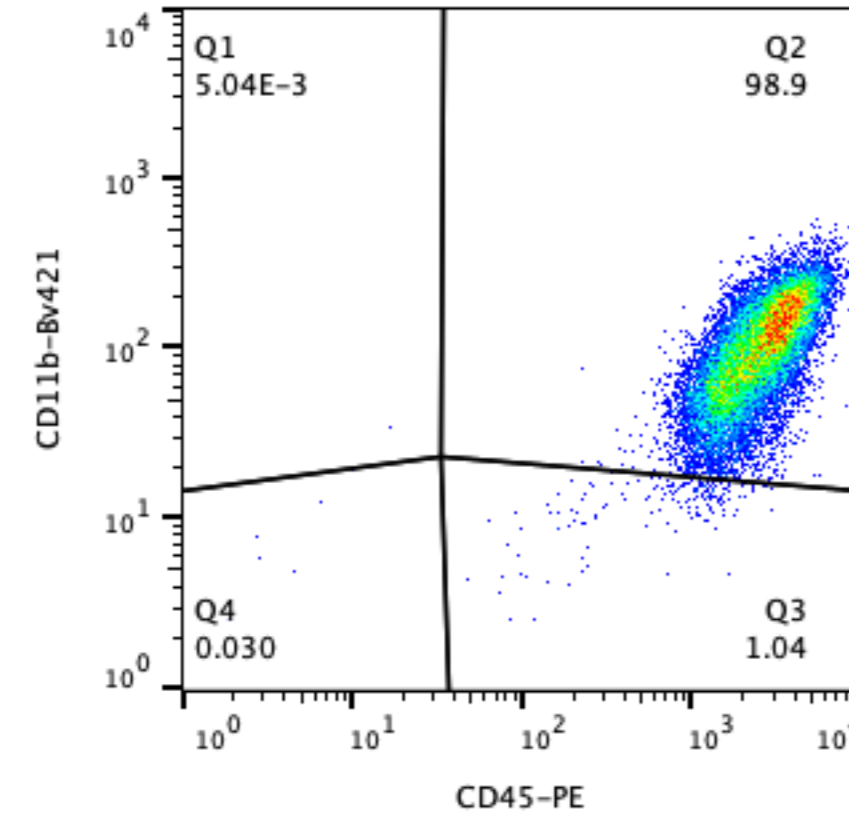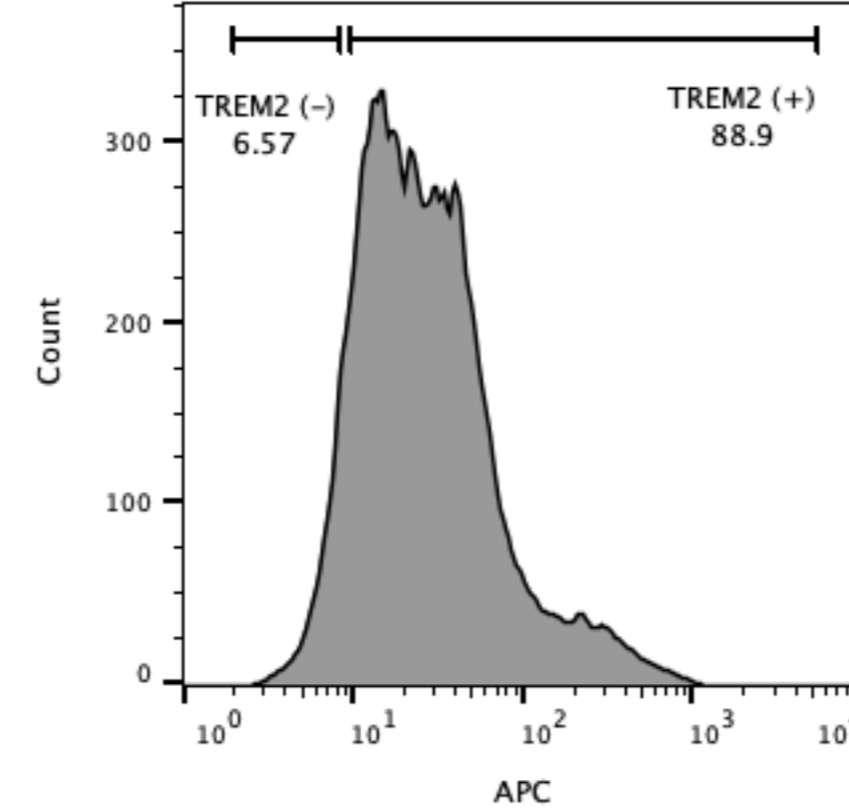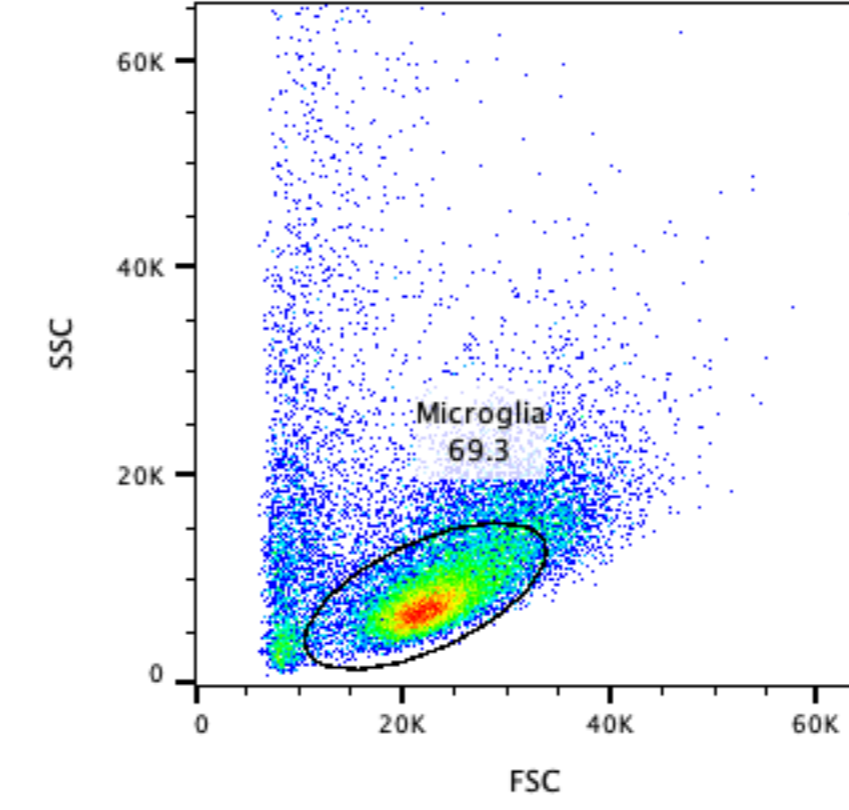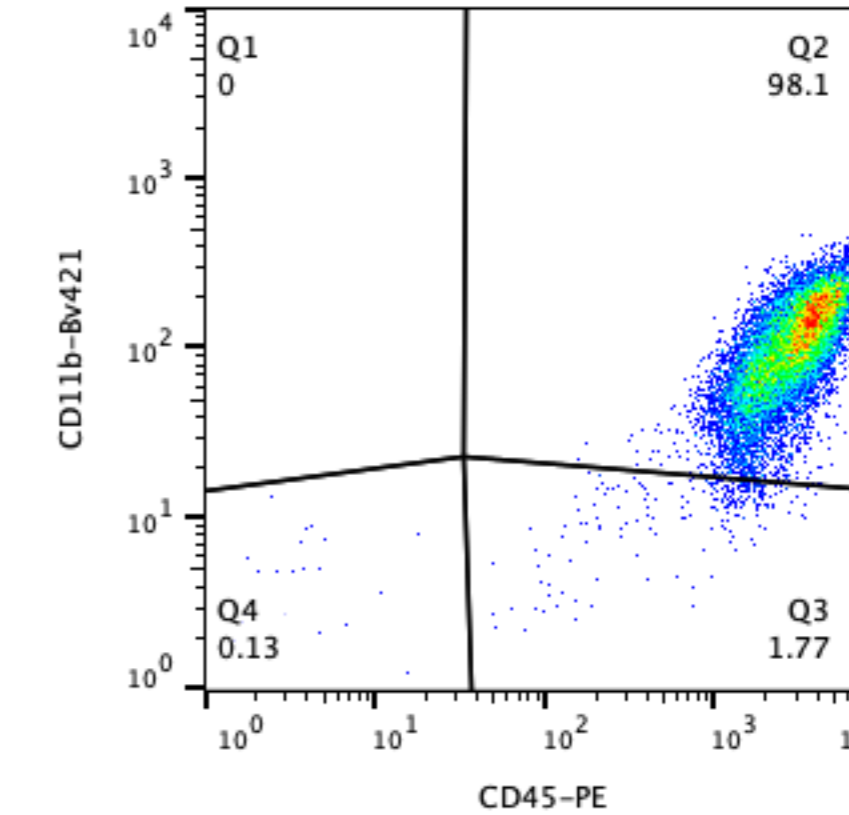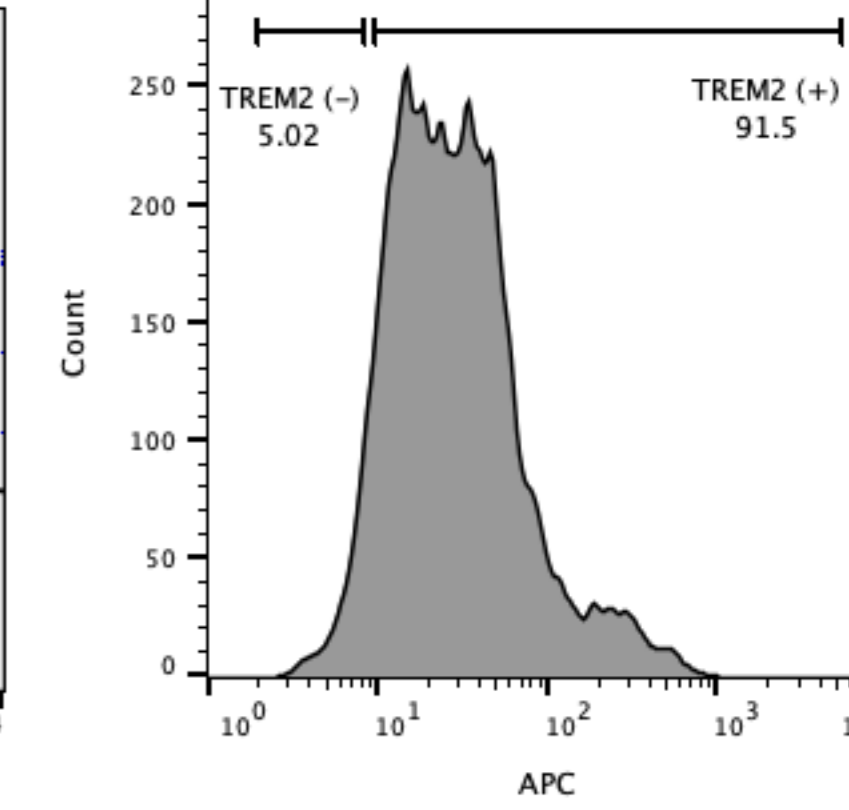

Supplement: S3 Data — (ZIP) [file pbio.3003320.s011.zip › S3_Data/S3_Data_B_iMGs_Characterization_FCS-files/Fig3_Bi_iMGs_CD45-and-TREM2_FCS-files/Fig3_Bi_iMGs_CD45-and-TREM2.pdf]

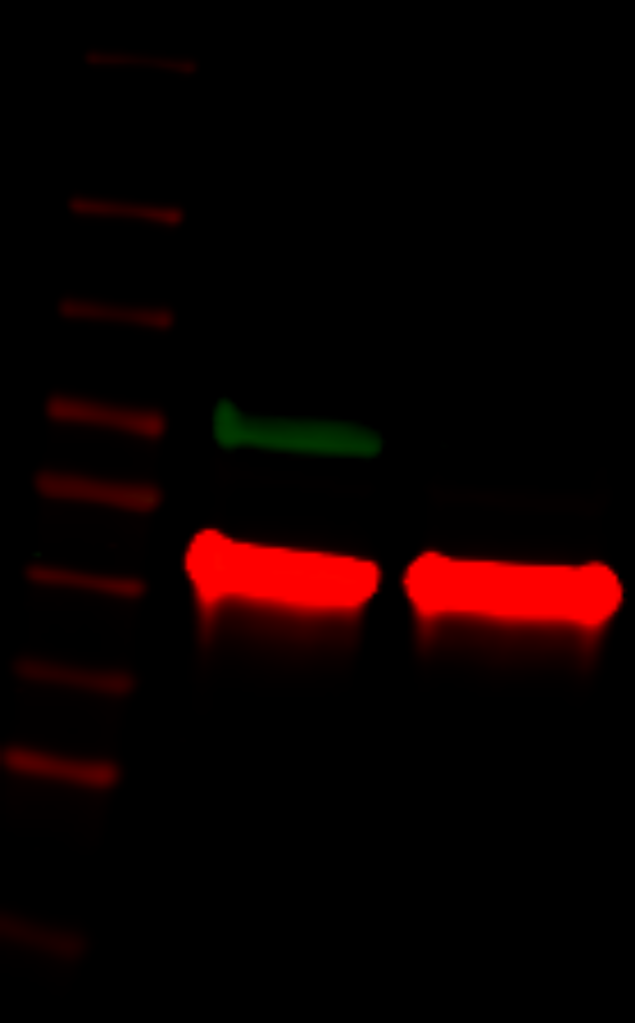

Supplement: S4 Data — (ZIP) [file pbio.3003320.s012.zip › S4_Data/S4_Data_H_UNC93B1.pdf]

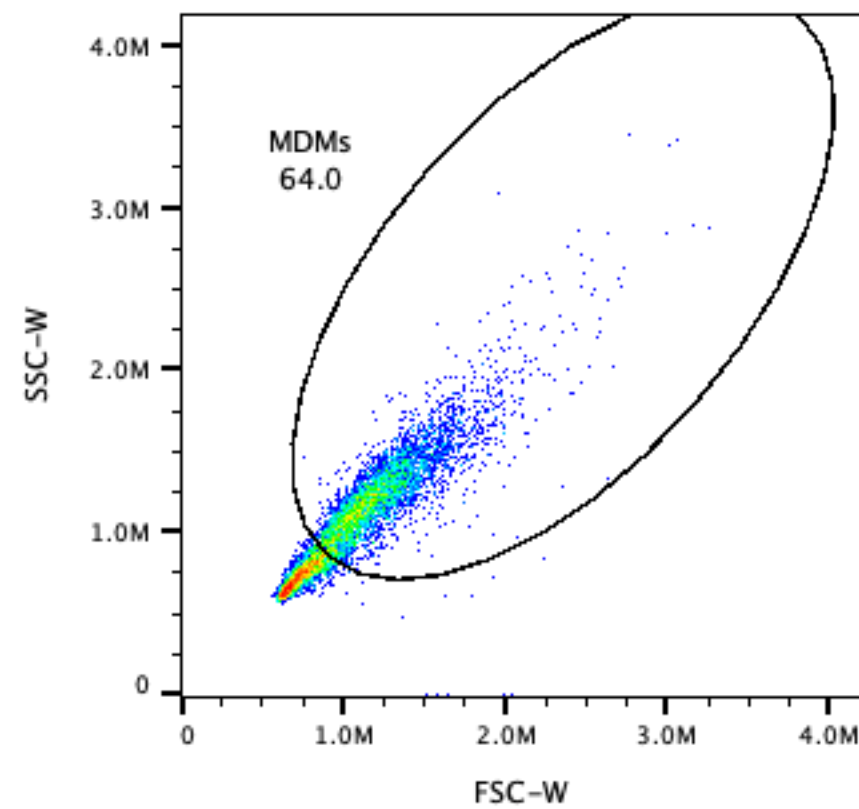

WT.fcs  
Ungated  
7368

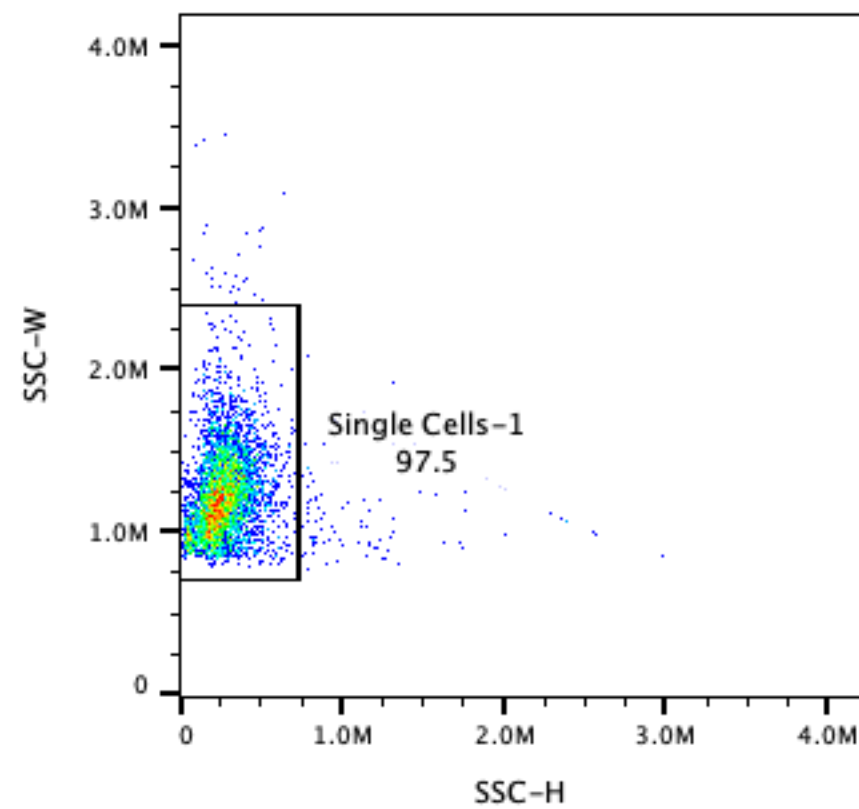

WT.fcs  
MDMs  
4717

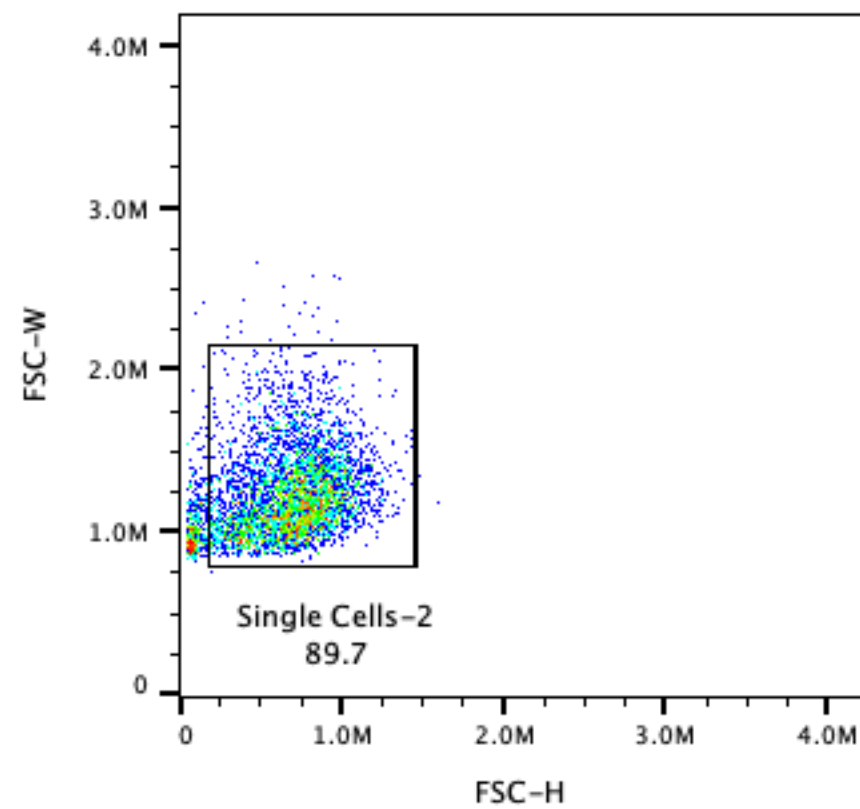

WT.fcs  
Single Cells-1  
4597

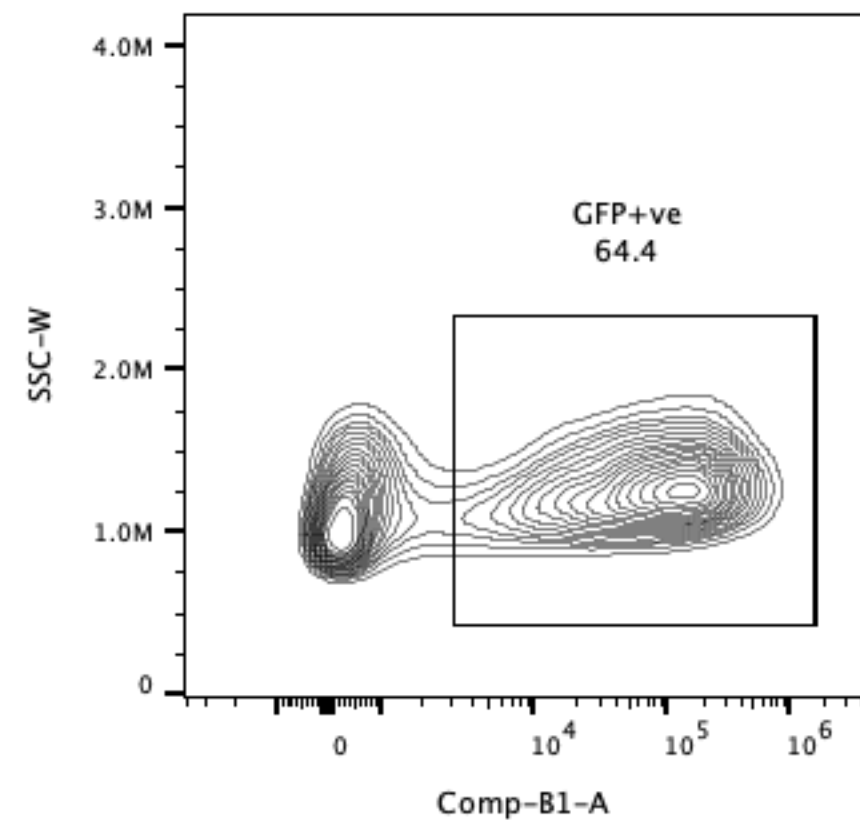

WT.fcs  
Single Cells-2  
4125

Supplement: S4 Data — (ZIP) [file pbio.3003320.s012.zip › S4_Data/S4_Data_A_MDMs_eGFP-infections_FCS-files/Fig4_A_MDMs_eGFP-infections-Fig4_A_MDMs_eGFP-infections_gating.pdf]

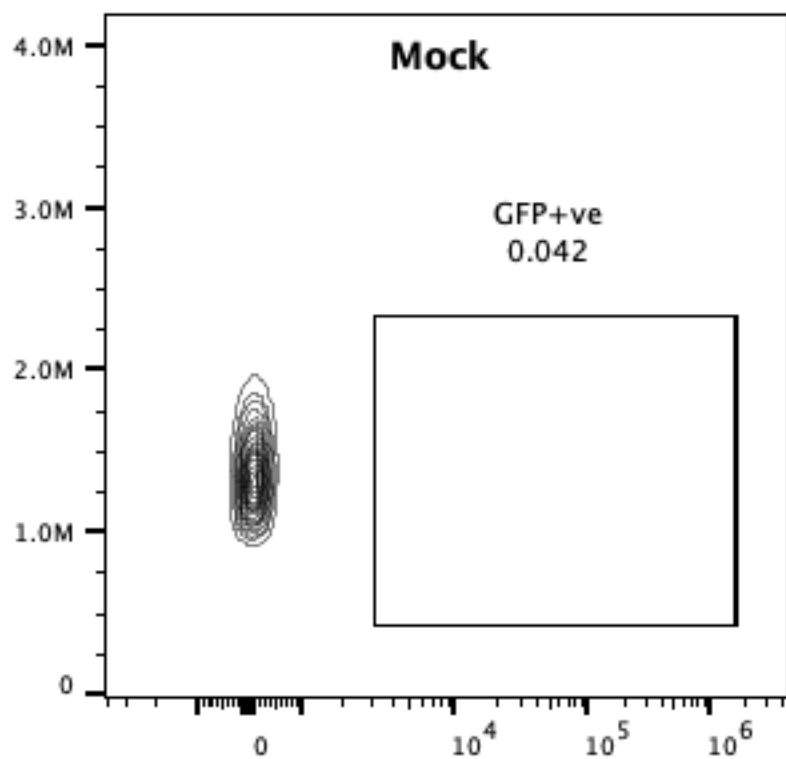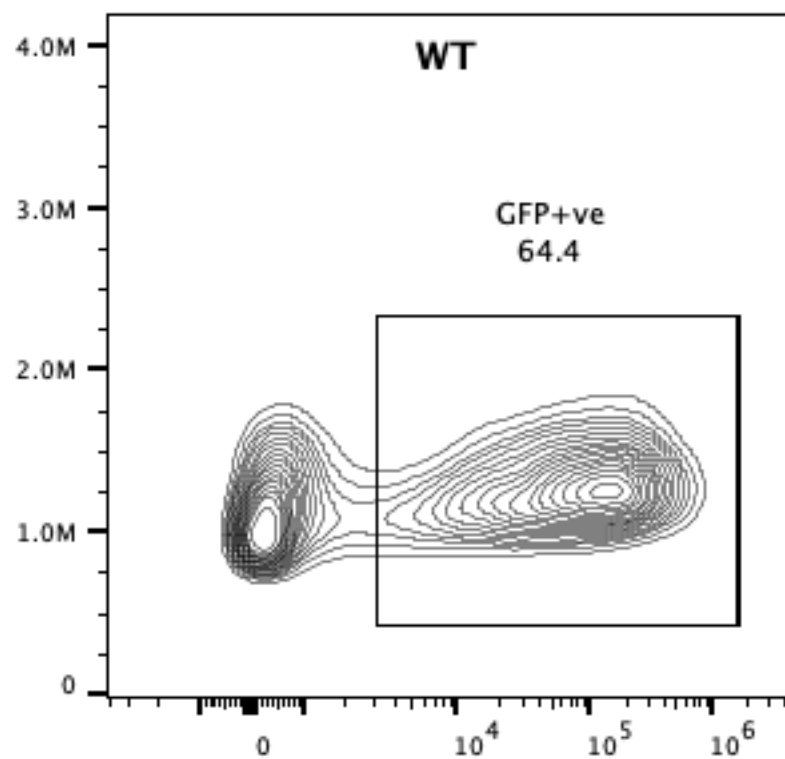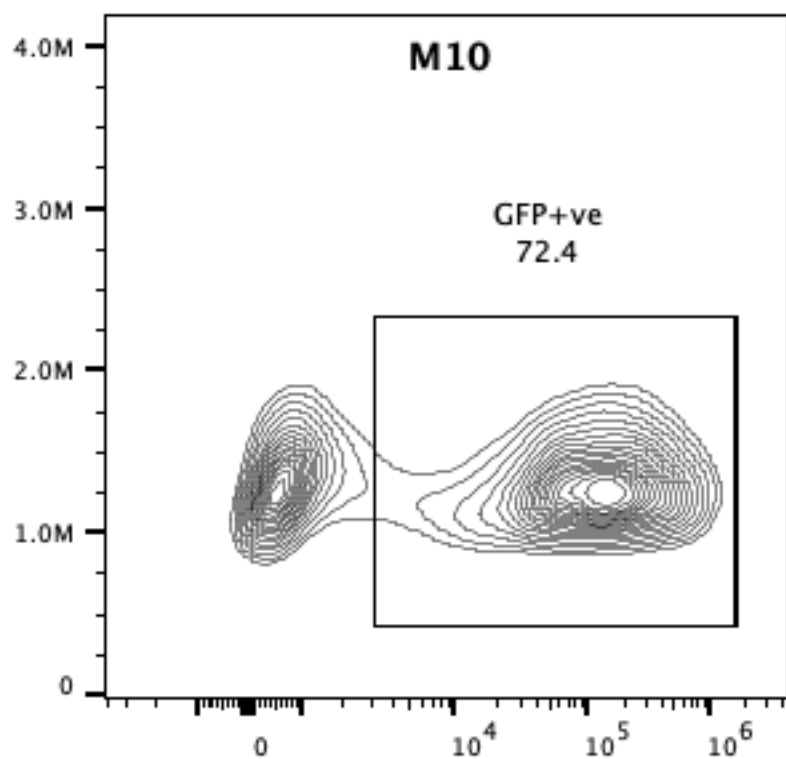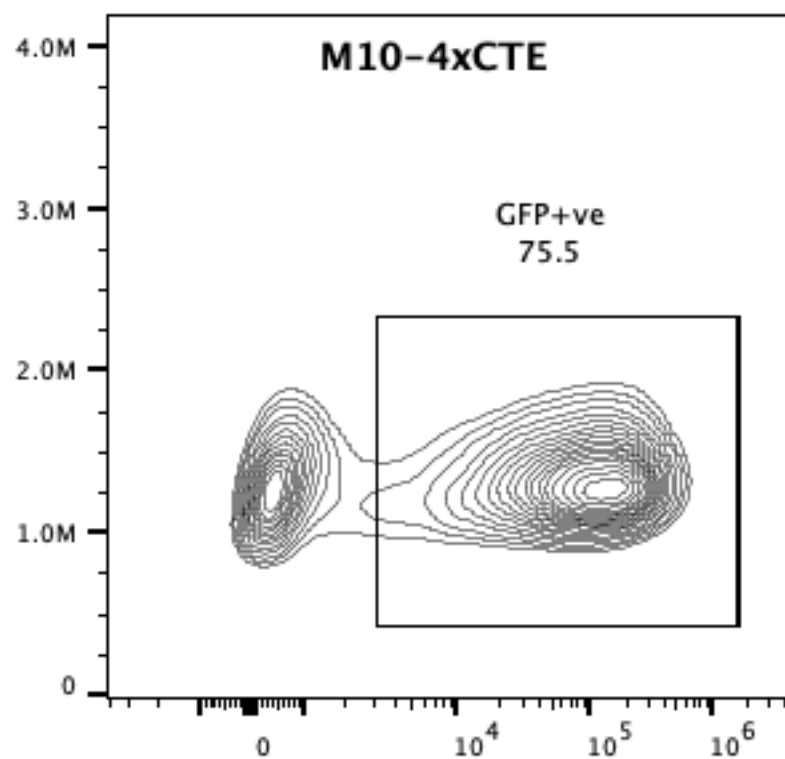

Supplement: S4 Data — (ZIP) [file pbio.3003320.s012.zip › S4_Data/S4_Data_A_MDMs_eGFP-infections_FCS-files/Fig4_A_MDMs_eGFP-infections-Fig4_A_MDMs_eGFP-infections_FCS.pdf]

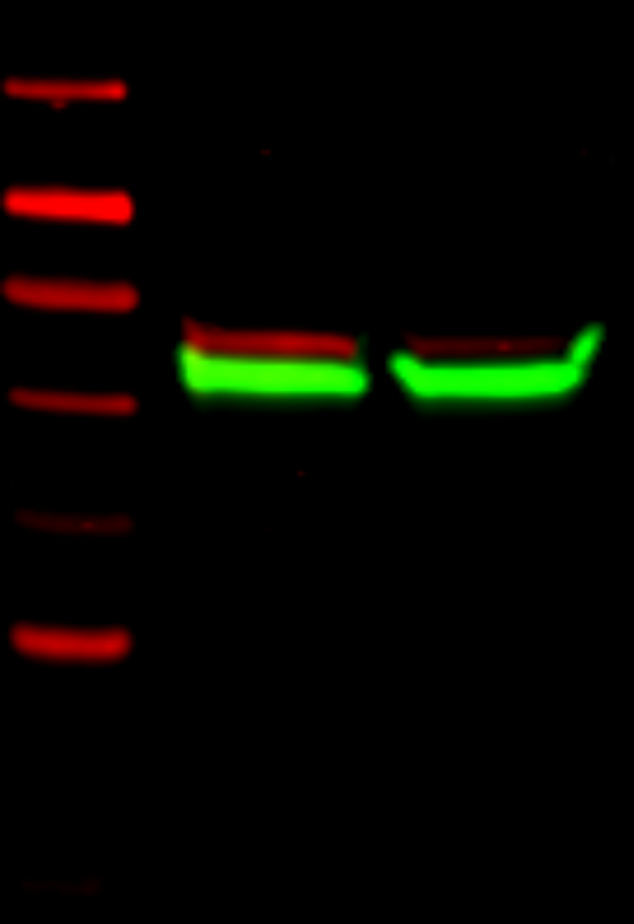

Supplement: S5 Data — (ZIP) [file pbio.3003320.s013.zip › S5_Data/S5_Data_B_CASP4_actin.pdf]

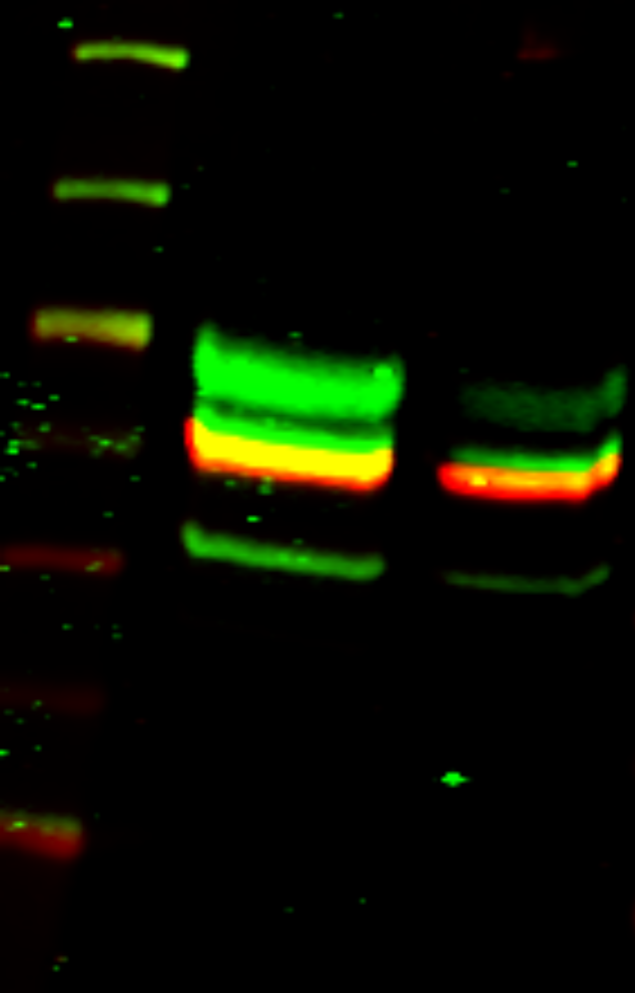

Supplement: S5 Data — (ZIP) [file pbio.3003320.s013.zip › S5_Data/S5_Data_B_AIM2.pdf]

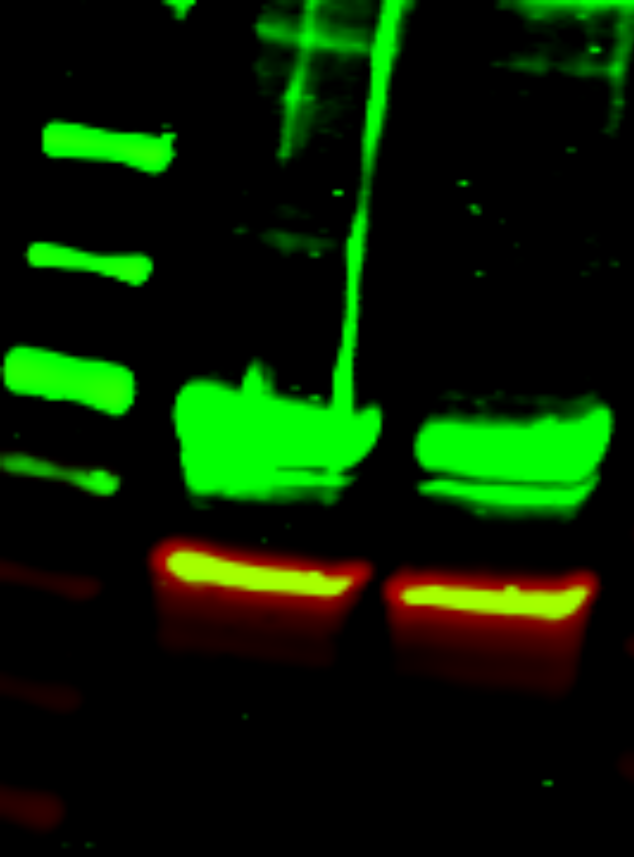

Supplement: S5 Data — (ZIP) [file pbio.3003320.s013.zip › S5_Data/S5_Data_B_NLRP1.pdf]

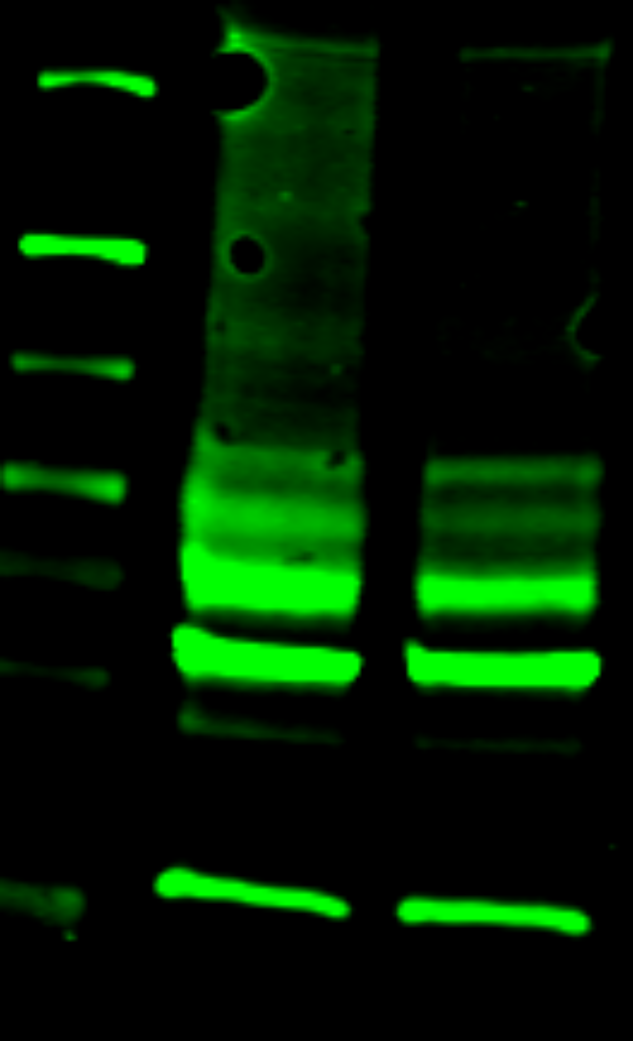

Supplement: S5 Data — (ZIP) [file pbio.3003320.s013.zip › S5_Data/S5_Data_B_NLRP3.pdf]

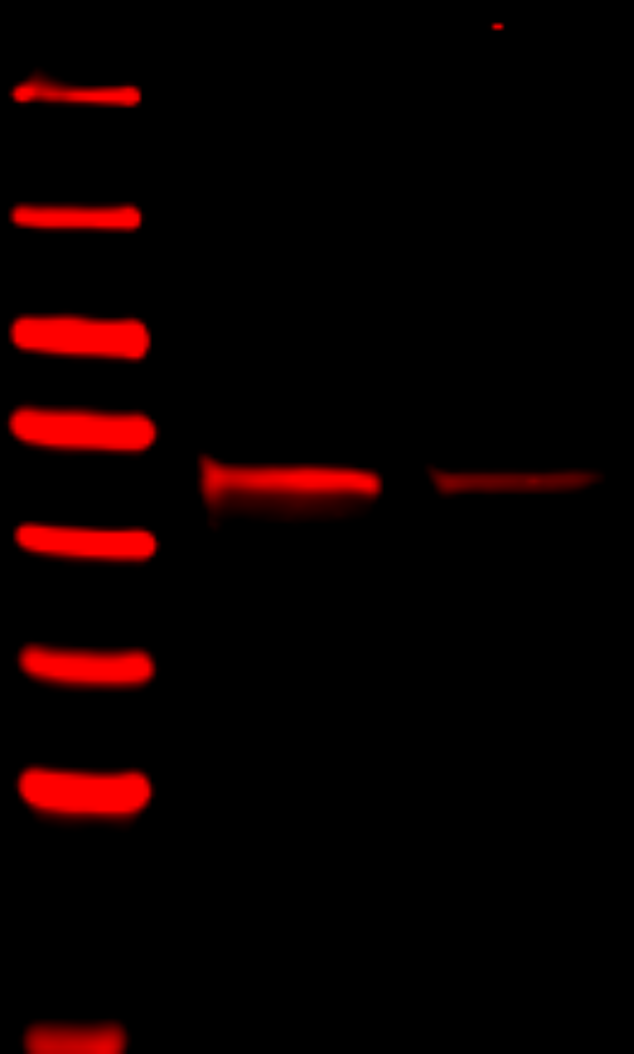

Supplement: S5 Data — (ZIP) [file pbio.3003320.s013.zip › S5_Data/S5_Data_B_CASP4.pdf]

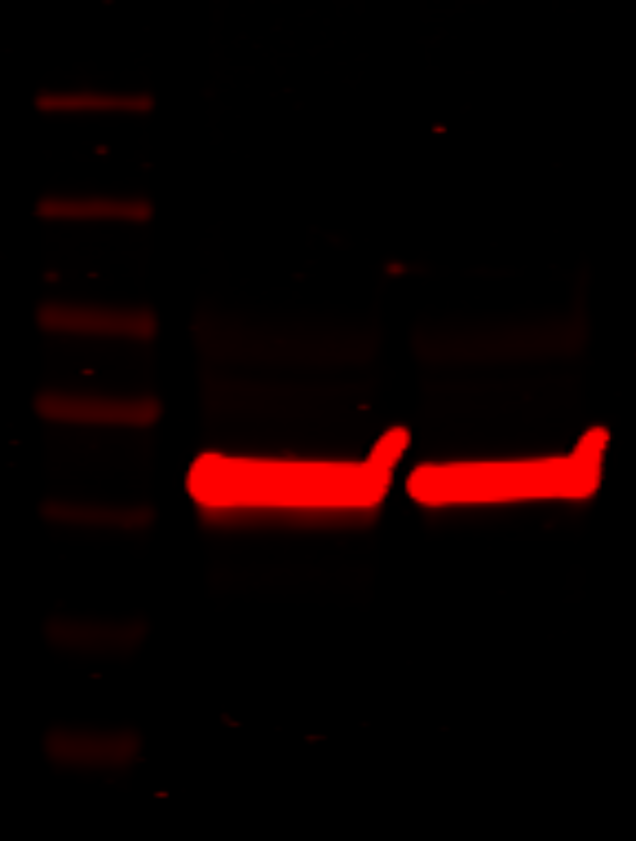

Supplement: S5 Data — (ZIP) [file pbio.3003320.s013.zip › S5_Data/S5_Data_B_CASP1_actin.pdf]

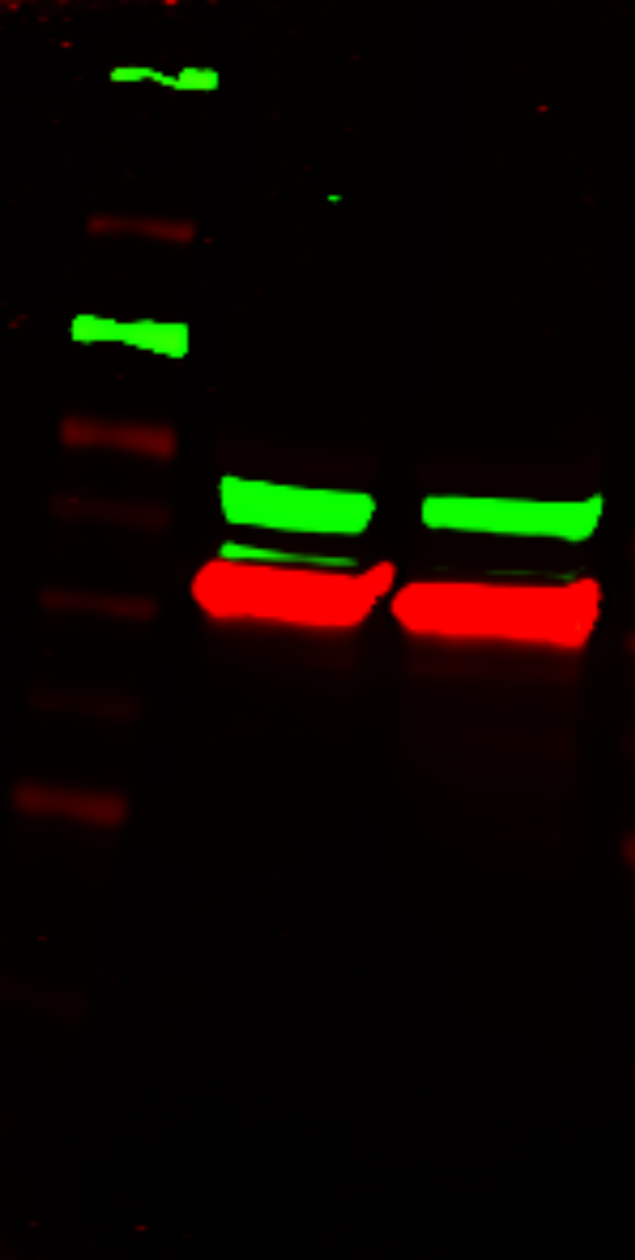

Supplement: S5 Data — (ZIP) [file pbio.3003320.s013.zip › S5_Data/S5_Data_B_CARD8.pdf]

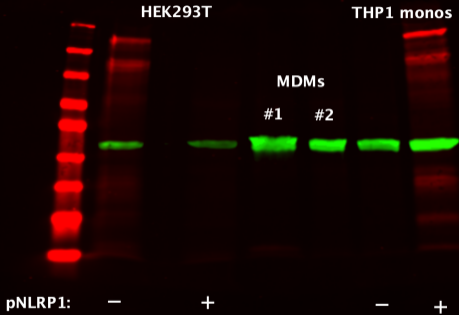

Supplement: S6 Data — (ZIP) [file pbio.3003320.s014.zip › S6_Data/S6_Data_A_NLRp1-overexpression.pdf]

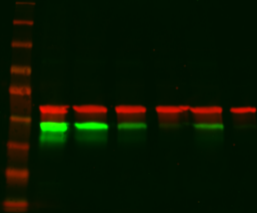

Supplement: S6 Data — (ZIP) [file pbio.3003320.s014.zip › S6_Data/S6_Data_Gii_THPNLRP1 shSTING.pdf]

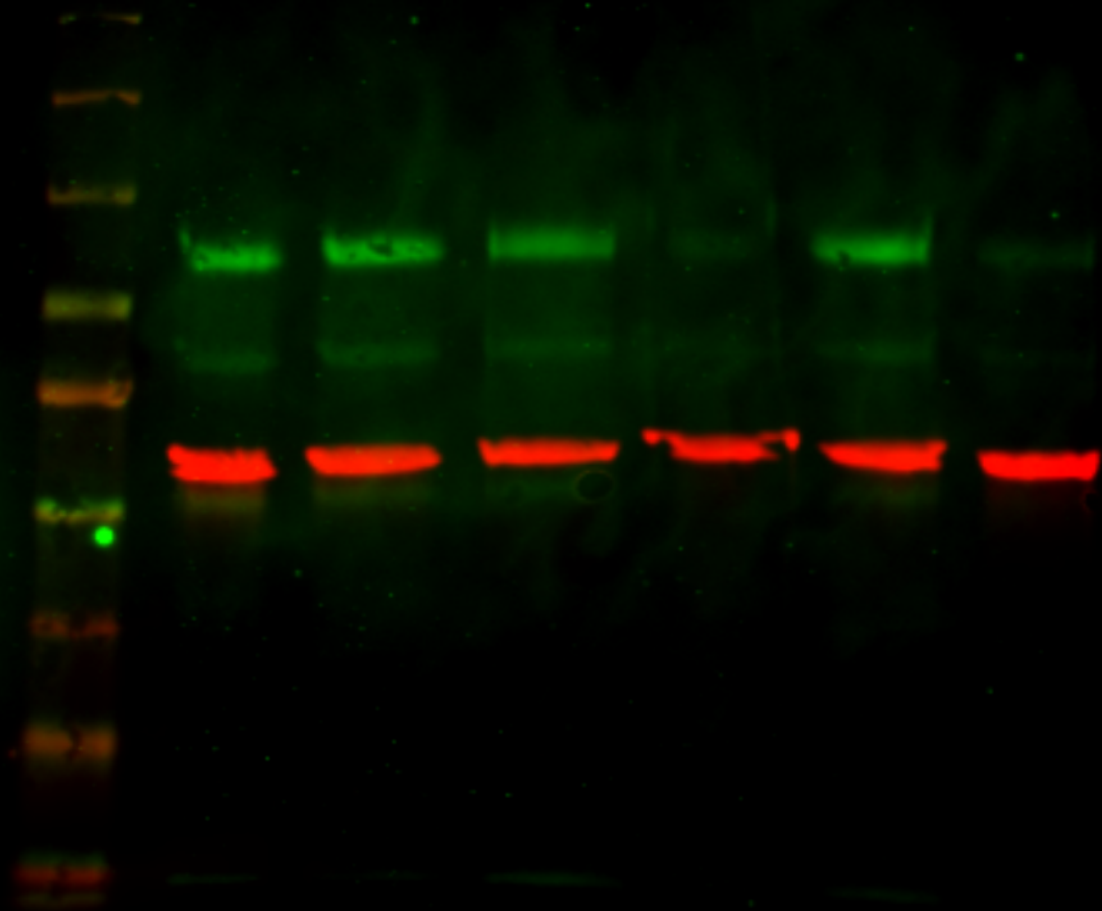

Supplement: S6 Data — (ZIP) [file pbio.3003320.s014.zip › S6_Data/S6_Data_Gi_THPNLRP1 shMAVS.pdf]

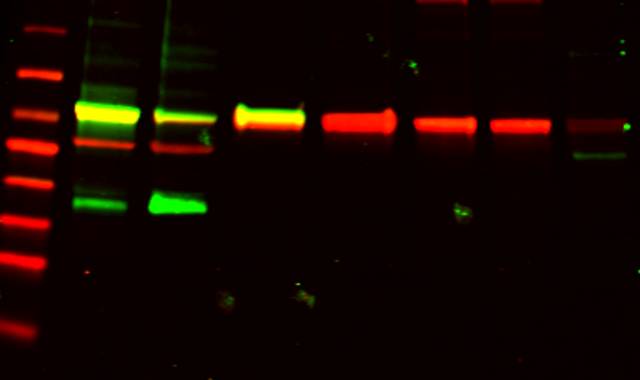

Supplement: S2 Supplementary Data — (ZIP) [file pbio.3003320.s017.zip › S2_Supplementary_data/FigS2_Ai_SAMHD1-and-pSAMHD1.pdf]

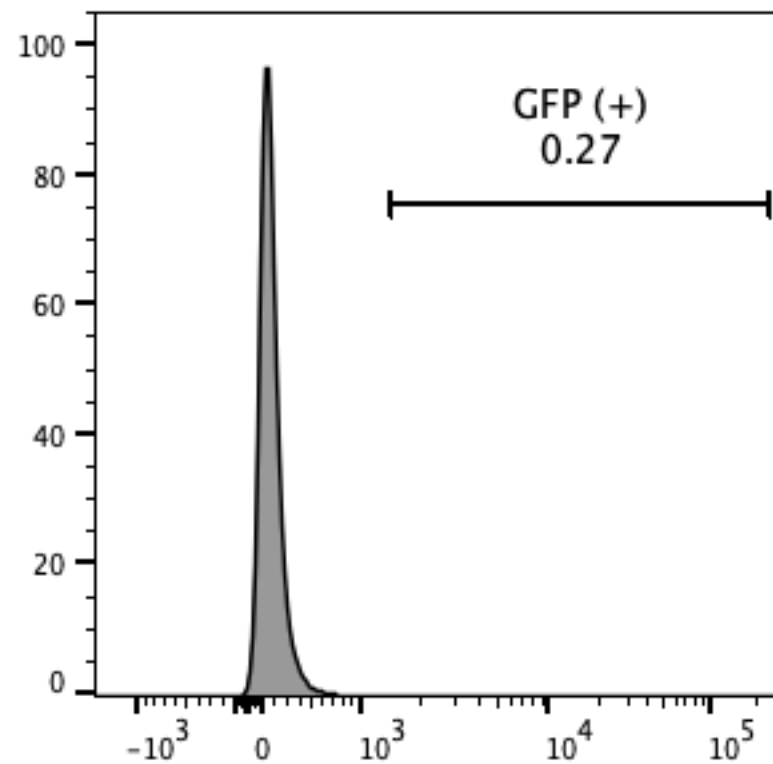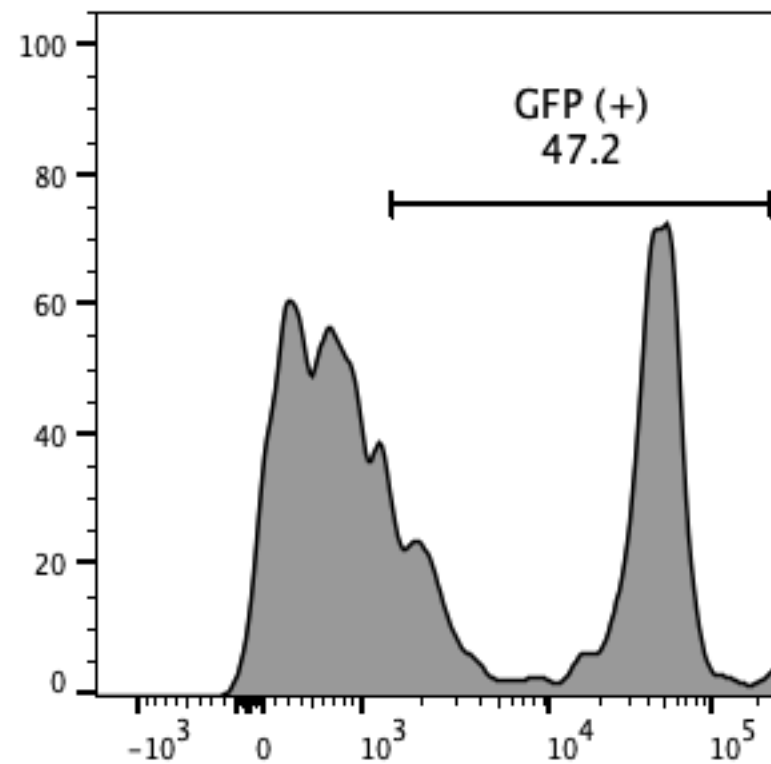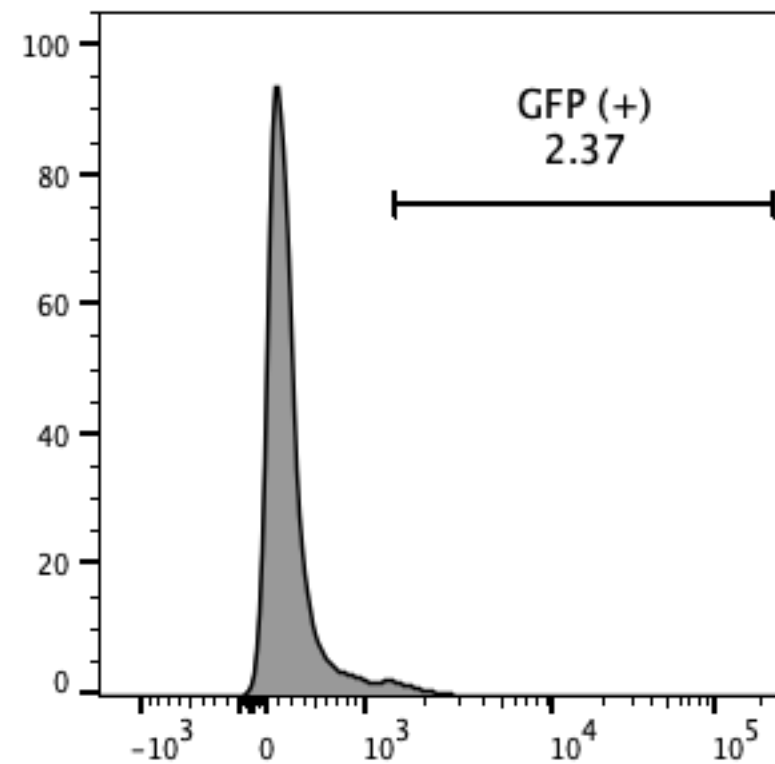

Supplement: S2 Supplementary Data — (ZIP) [file pbio.3003320.s017.zip › S2_Supplementary_data/FigS2_C_iMGs_infections_FCS-files/FigS2_C_iMGs_infections_FCS.pdf]

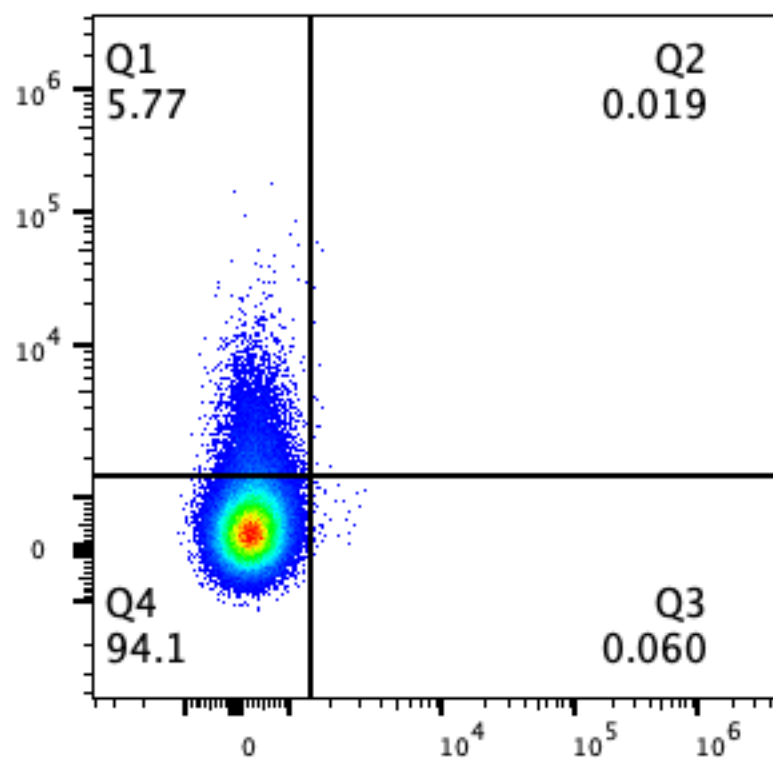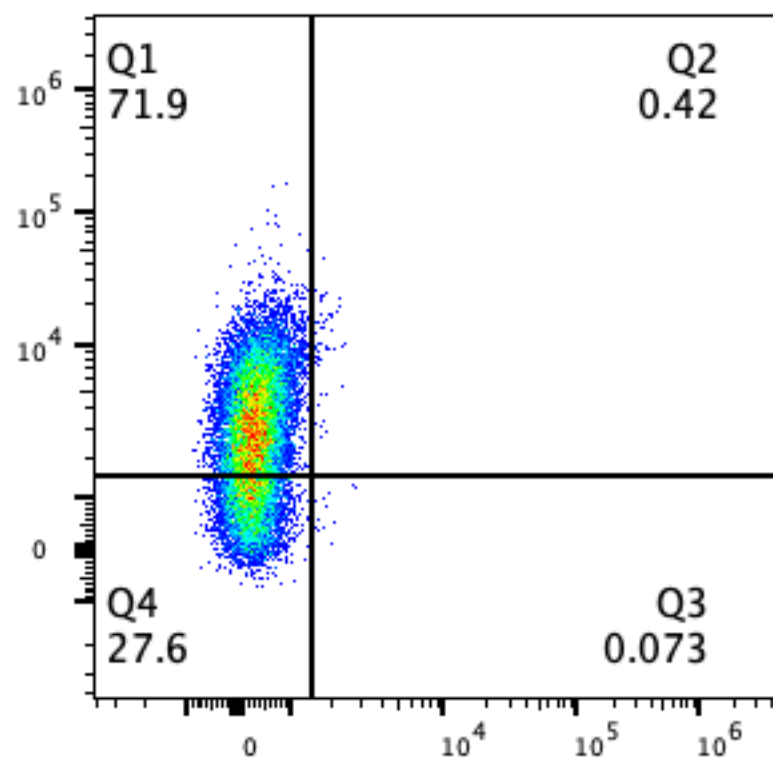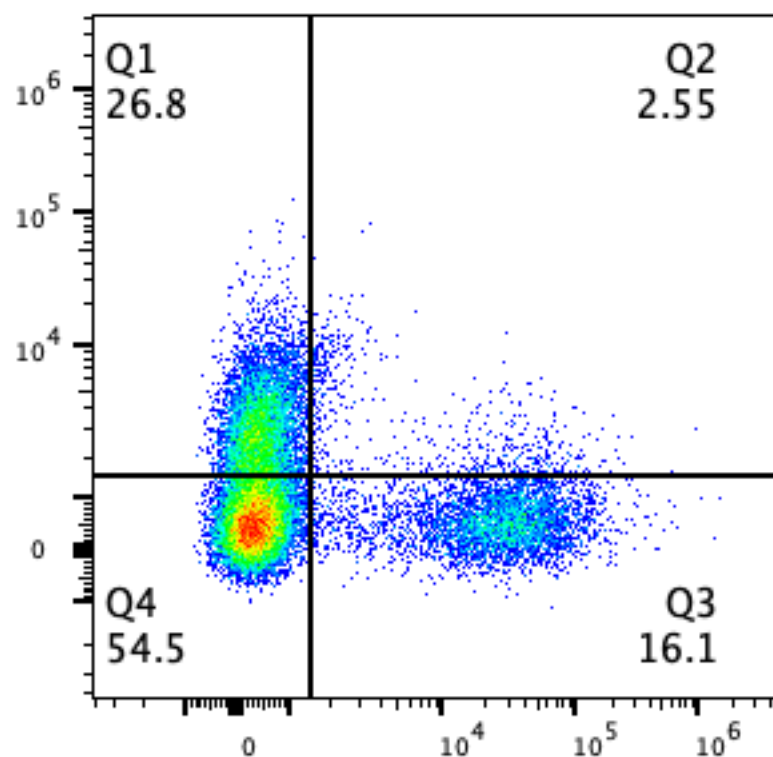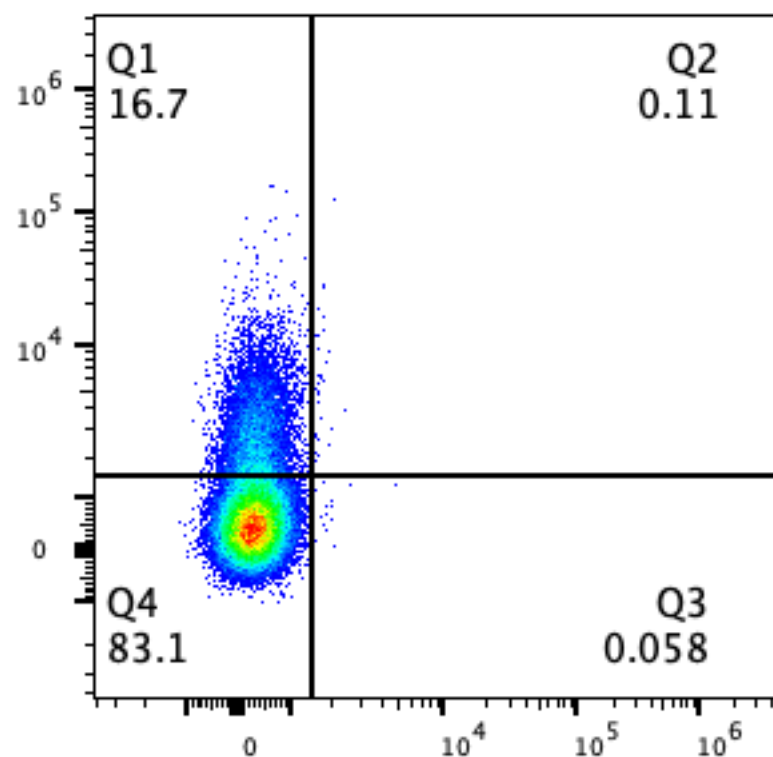

Supplement: S4 Supplementary Data — (ZIP) [file pbio.3003320.s019.zip › S4_Supplementary_data/FigS5_A_FLICA_FCS-files/FigS5_A_FLICA.pdf]

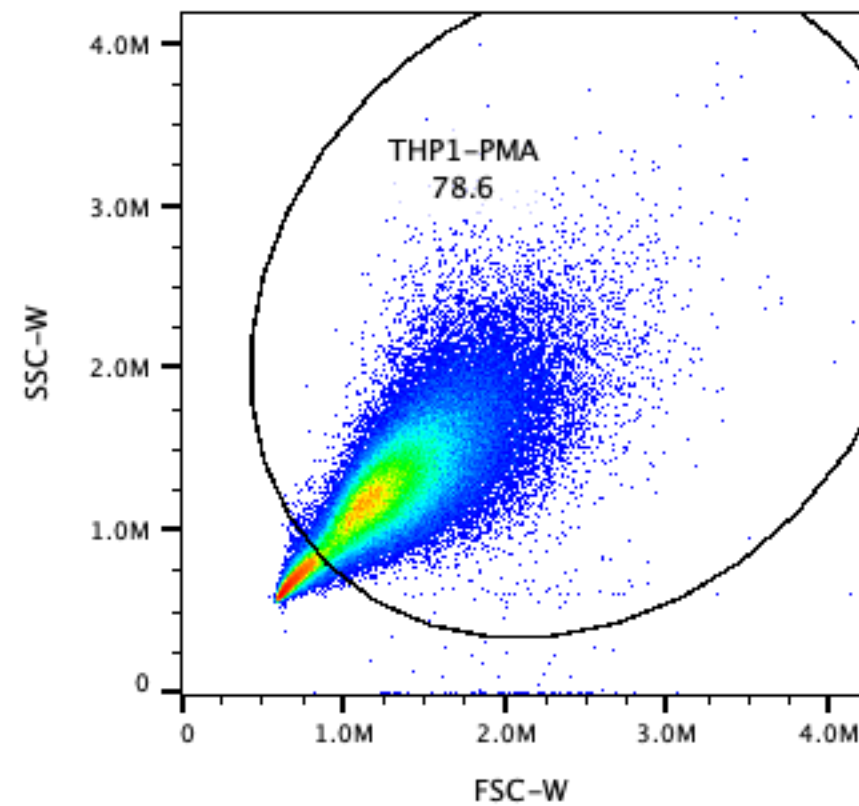

Mock.fcs  
Ungated  
126712

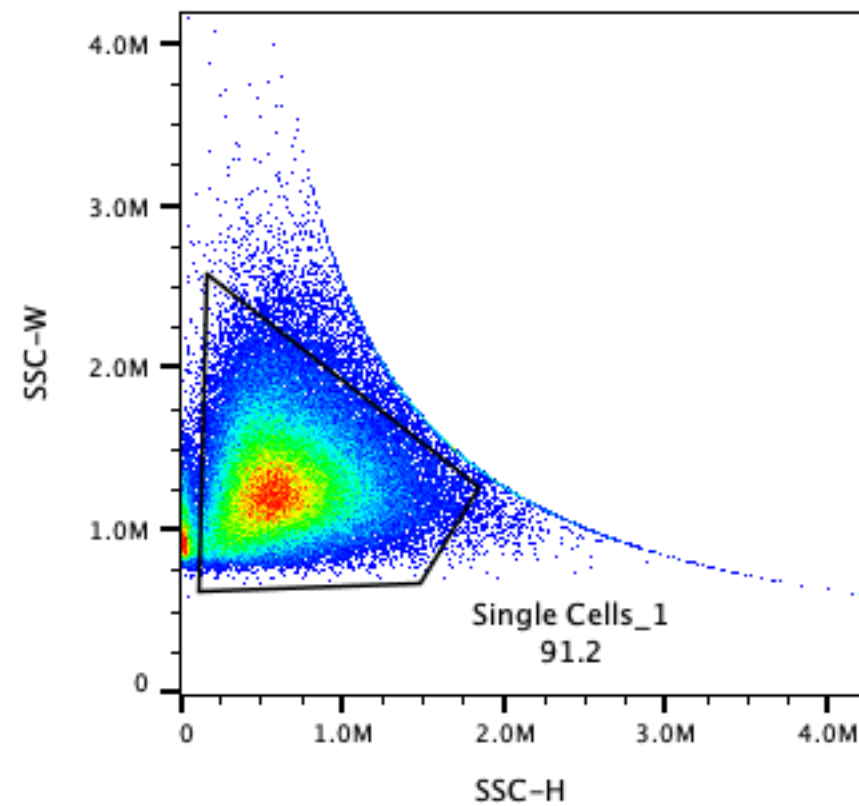

Mock.fcs  
THP1-PMA  
99605

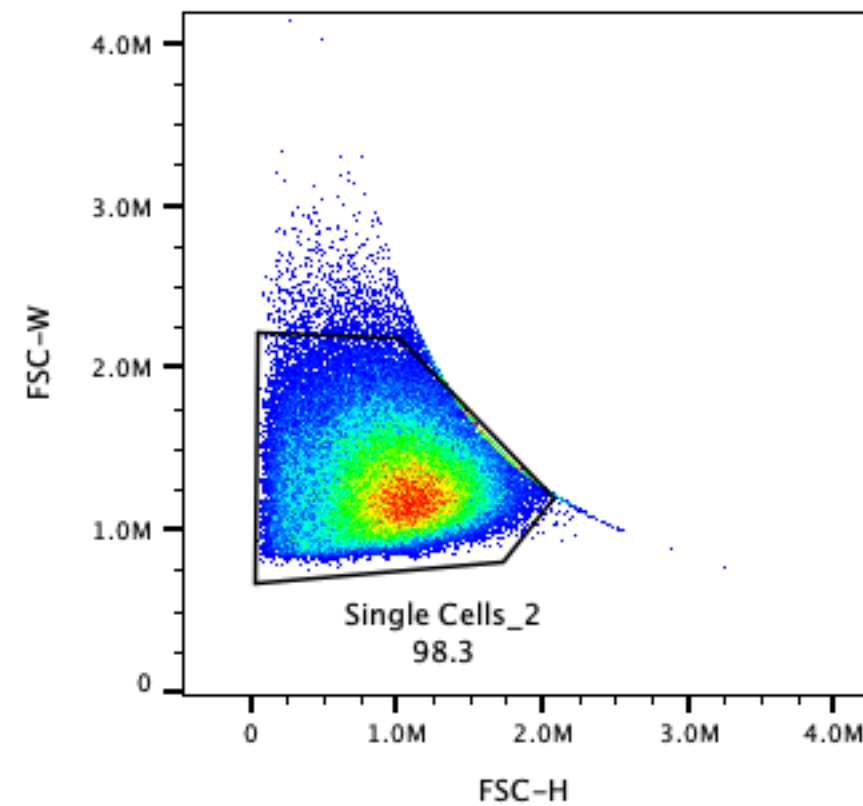

Mock.fcs  
Single Cells\_1  
90868

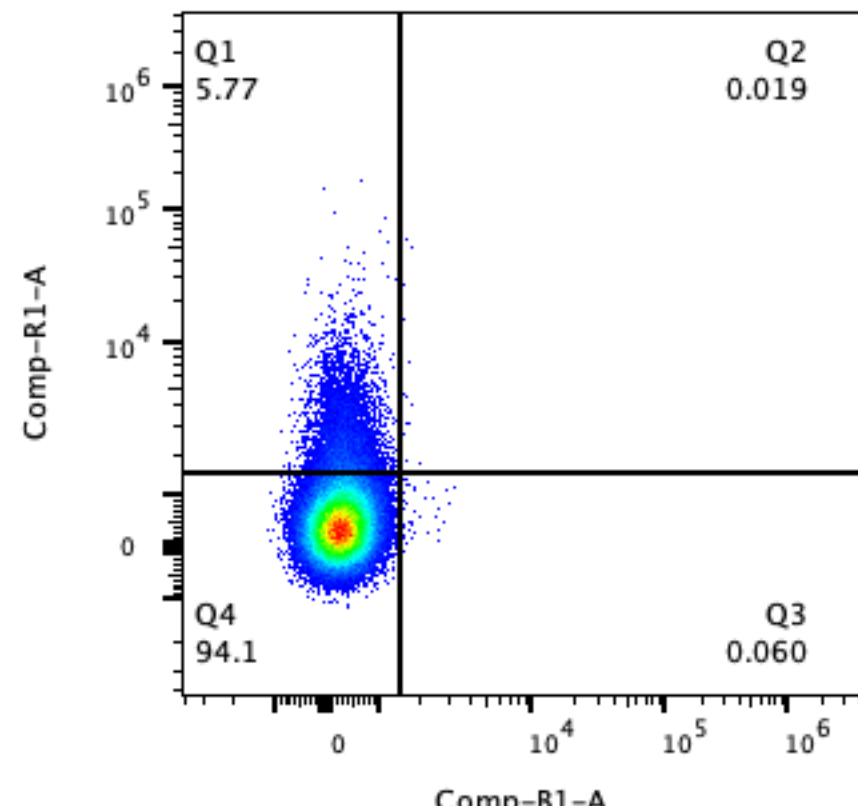

Mock.fcs  
Single Cells\_2  
89341

Supplement: S4 Supplementary Data — (ZIP) [file pbio.3003320.s019.zip › S4_Supplementary_data/FigS5_A_FLICA_FCS-files/FigS5_A_FLICA_gating.pdf]

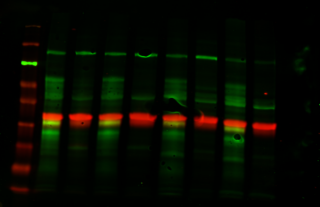

Supplement: S4 Supplementary Data — (ZIP) [file pbio.3003320.s019.zip › S4_Supplementary_data/FigS5_F_CARD8.pdf]

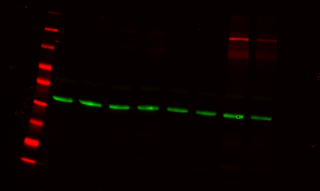

Supplement: S4 Supplementary Data — (ZIP) [file pbio.3003320.s019.zip › S4_Supplementary_data/FigS5_G_CARD8-NLRP1.pdf]
