# Supplementary material for: Expression of intron-containing HIV-1 RNA induces NLRP1 inflammasome activation in myeloid cells
Source: PLoS Biol. 2025 Sep 8;23(9):e3003320. doi: 10.1371/journal.pbio.3003320 (PMC12416851; doi:10.1371/journal.pbio.3003320)
Supplement: S1 Raw Images — (PDF) [file pbio.3003320.s007.pdf]

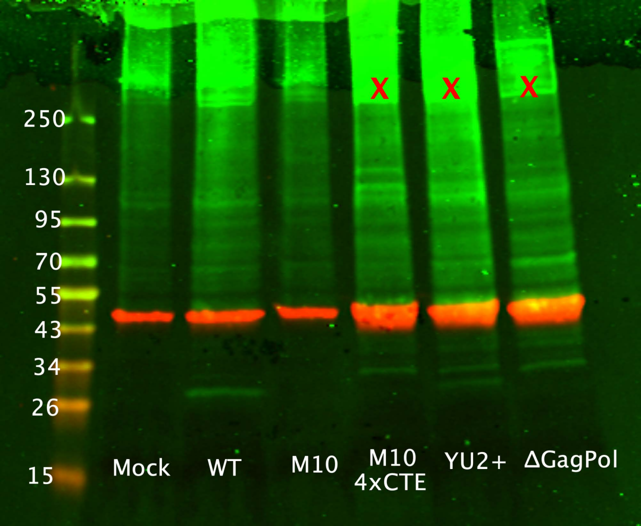

250
130
95
70
55
43
34
26
15
X
X
X
Mock
WT
M10
M10
4xCTE
YU2+
ΔGagPol

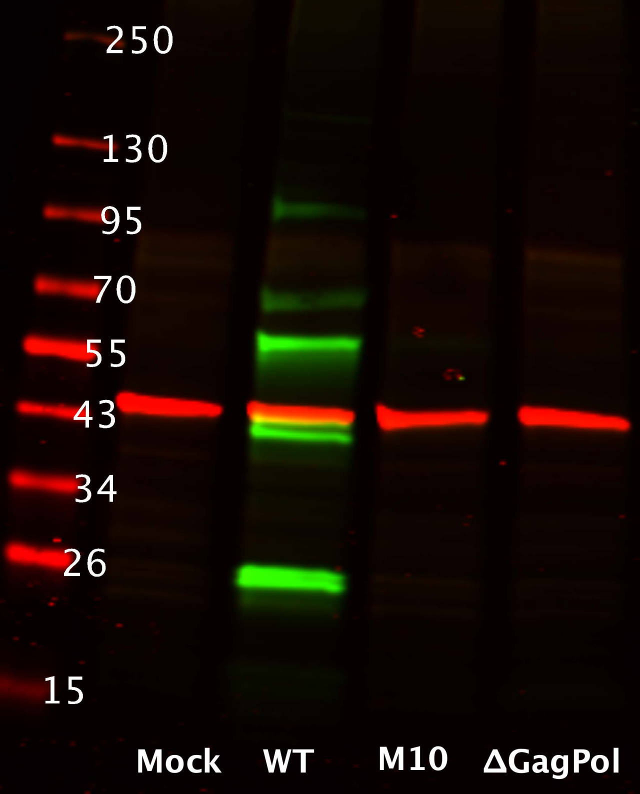
250
130
95
70
55
43
34
26
15
Mock
WT
M10
ΔGagPol

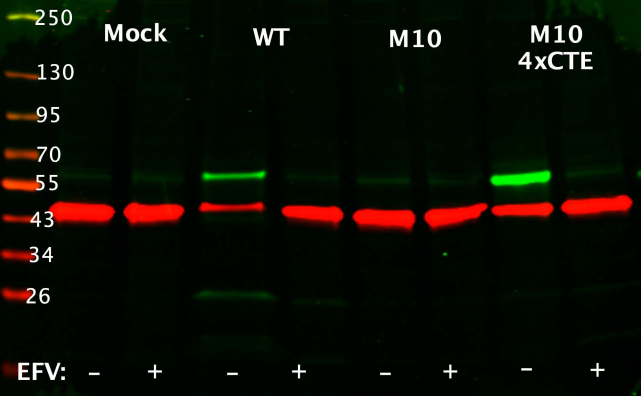
250
130
95
70
55
43
34
26
Mock
WT
M10
M10
4xCTE
EFV:
–
+
–
+
–
+
–
+

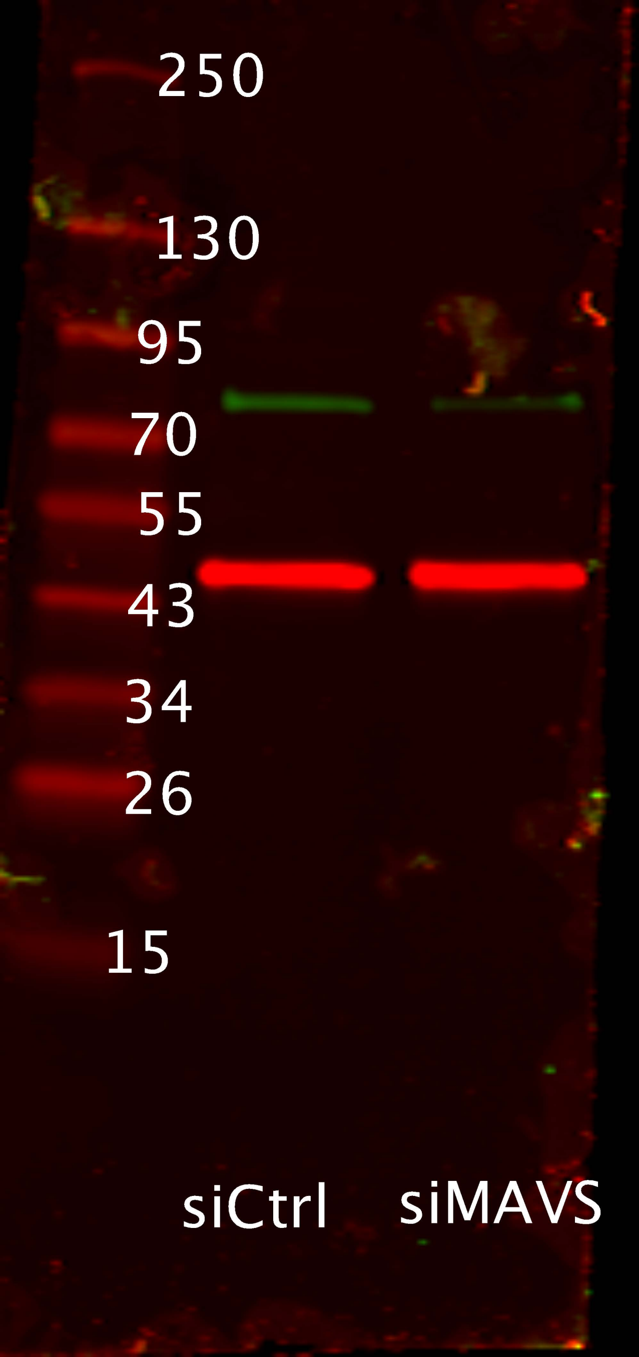
250
130
95
70
55
43
34
26
15
siCtrl
siMAVS

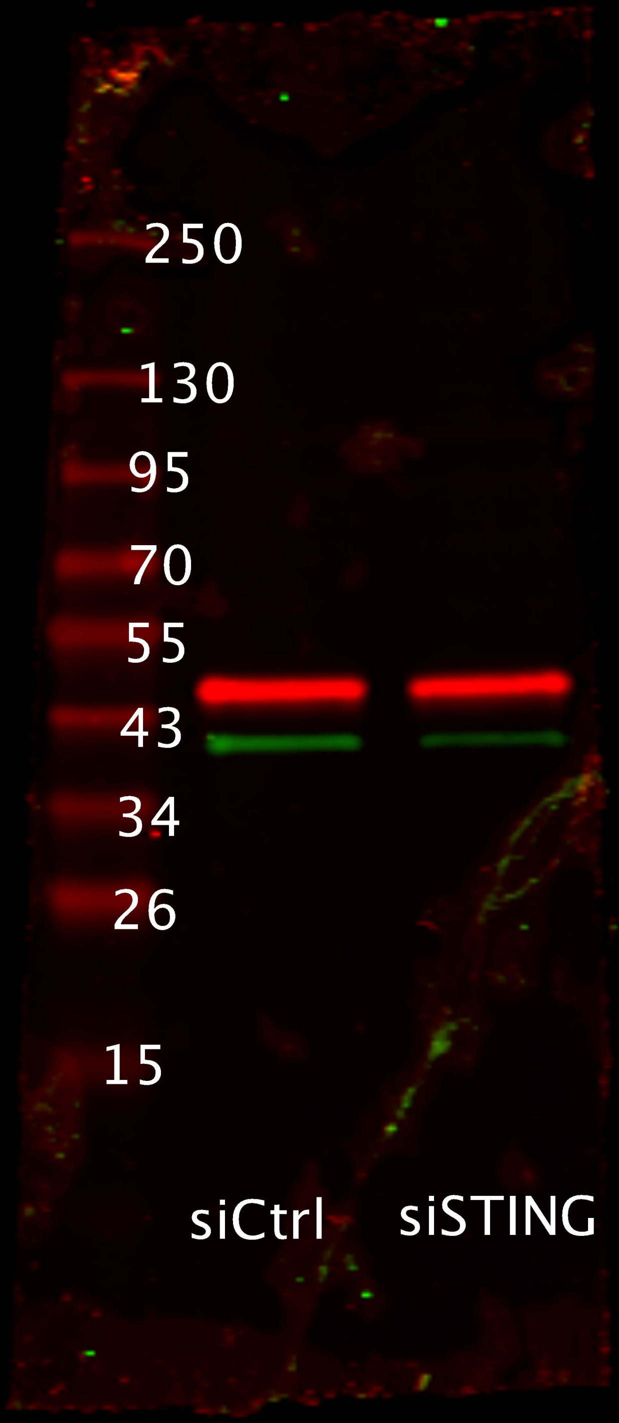
250
130
95
70
55
43
34
26
15
siCtrl
siSTING

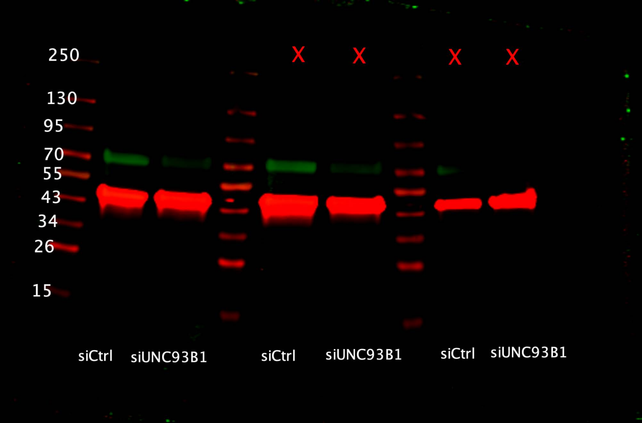
250
130
95
70
55
43
34
26
15
X
X
X
X
siCtrl
siUNC93B1
siCtrl
siUNC93B1
siCtrl
siUNC93B1

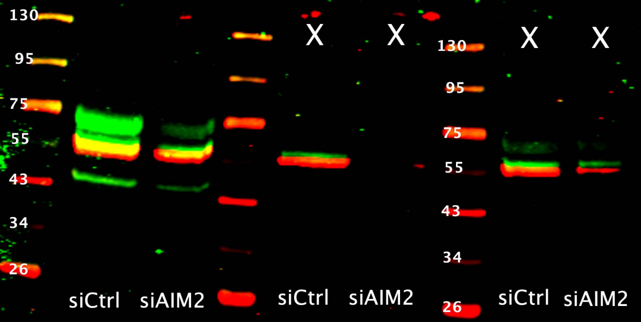
130
95
75
55
43
34
26
X
X
130
95
75
55
43
34
26
X
X
siCtrl
siAIM2
siCtrl
siAIM2
siCtrl
siAIM2

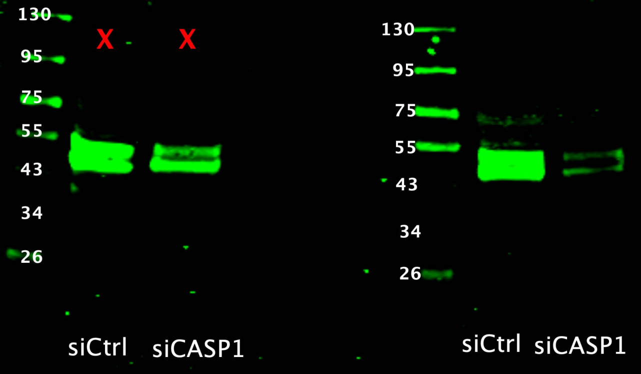

130
95
75
55
43
34
26
X
X
siCtrl
siCASP1
130
95
75
55
43
34
26
siCtrl
siCASP1

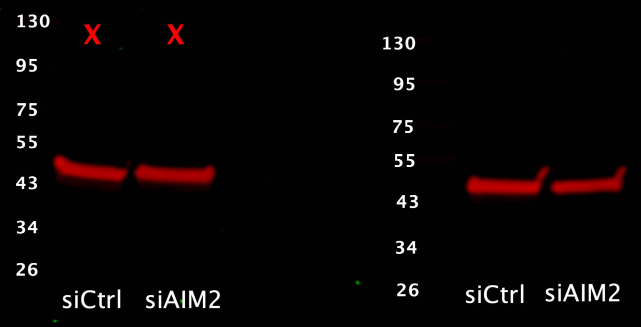
130
X
X
95
75
55
43
34
26
siCtrl
siAIM2
130
95
75
55
43
34
26
siCtrl
siAIM2

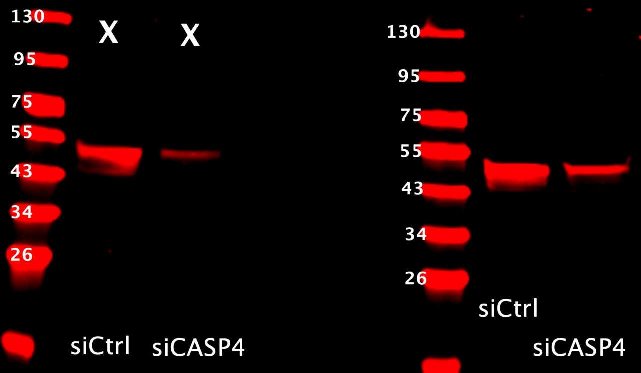
130
95
75
55
43
34
26
X
X
siCtrl
siCASP4
130
95
75
55
43
34
26
siCtrl
siCASP4

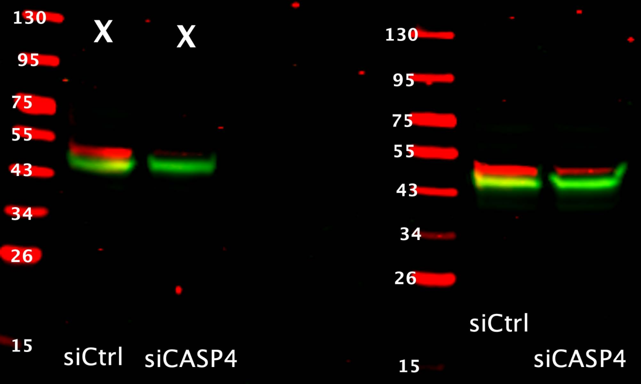
130
95
75
55
43
34
26
15
X
X
siCtrl
siCASP4
130
95
75
55
43
34
26
15
siCtrl
siCASP4

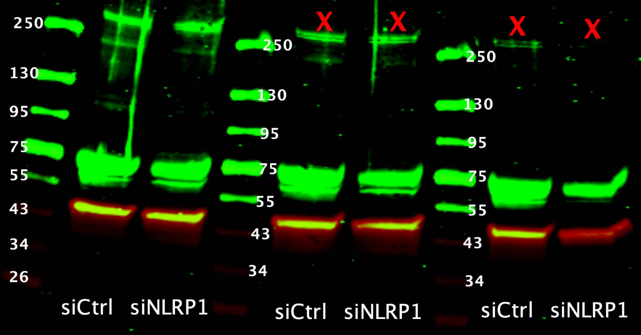

250
130
95
75
55
43
34
26
siCtrl
siNLRP1
X
X
250
130
95
75
55
43
34
siCtrl
siNLRP1
X
X
250
130
95
75
55
43
34
siCtrl
siNLRP1

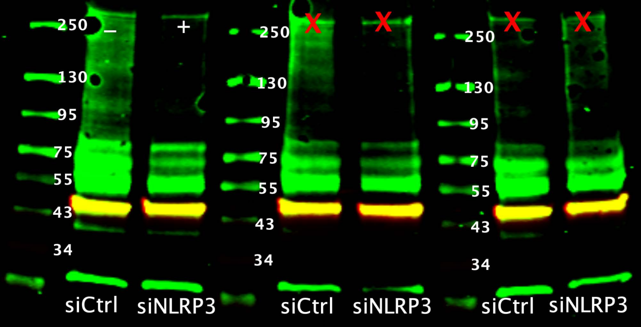
250
130
95
75
55
43
34
–
+
X
X
X
X
siCtrl
siNLRP3
siCtrl
siNLRP3
siCtrl
siNLRP3

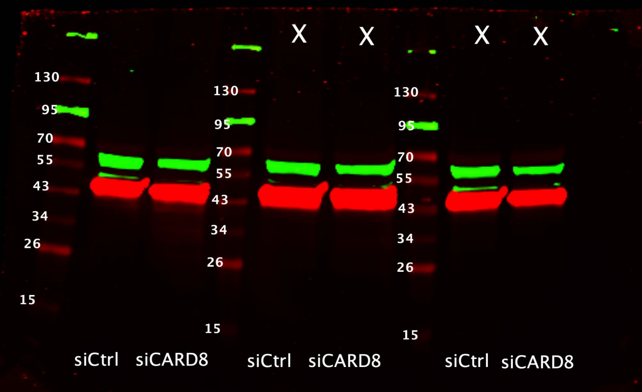
X
X
X
X
130
95
70
55
43
34
26
15
130
95
70
55
43
34
26
15
130
95
70
55
43
34
26
15
siCtrl
siCARD8
siCtrl
siCARD8
siCtrl
siCARD8

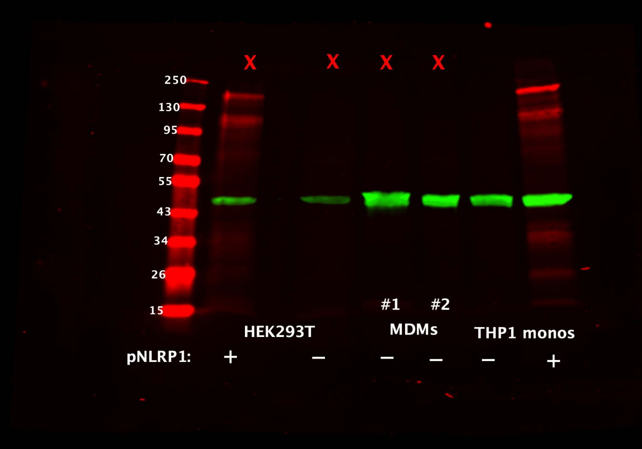
X
X
X
X
250
130
95
70
55
43
34
26
15
#1
#2
HEK293T
MDMs
THP1 monos
pNLRP1:
+
−
−
−
−
+

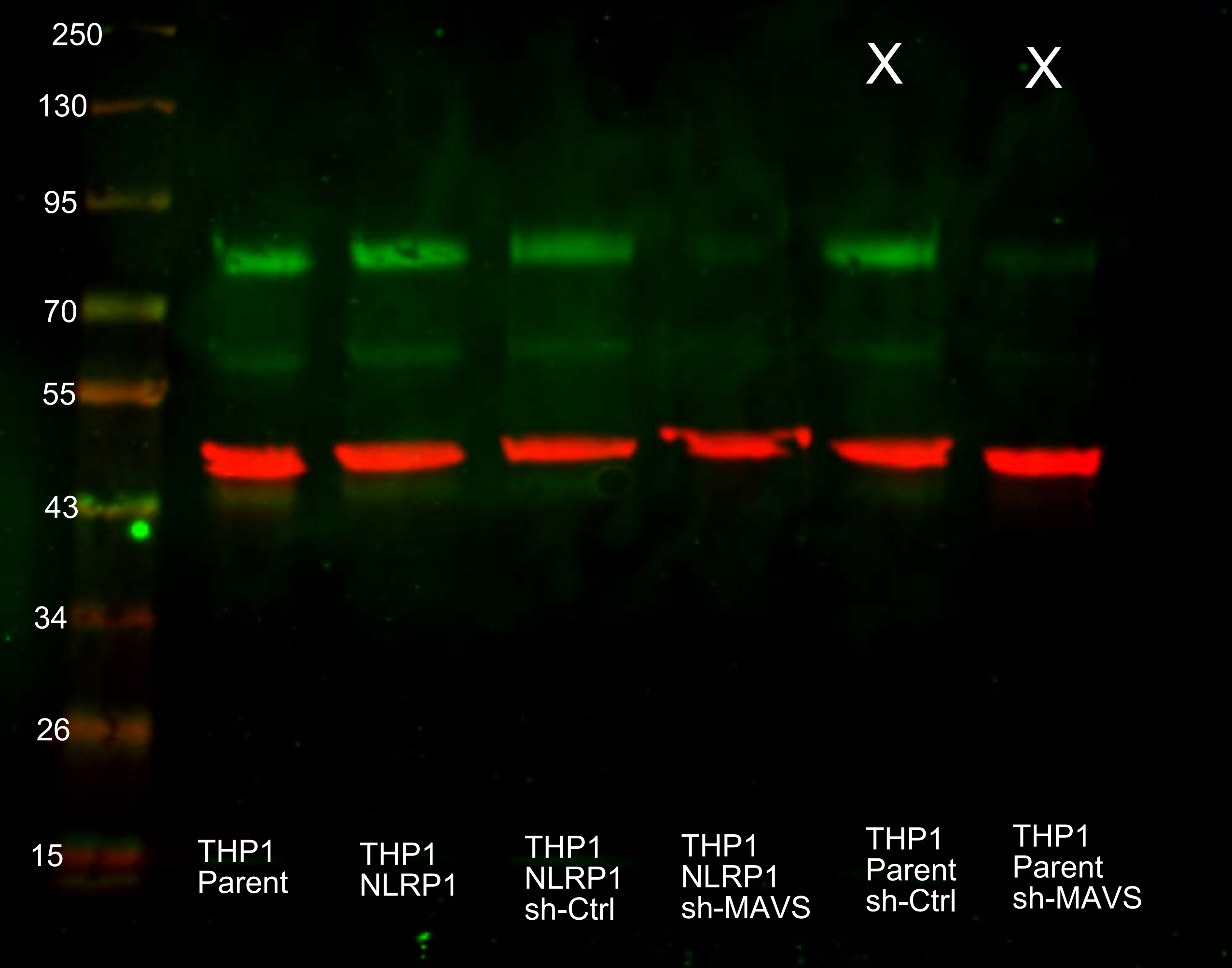
250
130
95
70
55
43
34
26
15
X
X
THP1 Parent
THP1 NLRP1
THP1 NLRP1 sh-Ctrl
THP1 NLRP1 sh-MAVS
THP1 Parent sh-Ctrl
THP1 Parent sh-MAVS

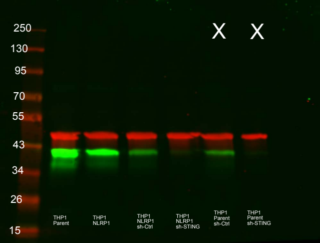

250
130
95
70
55
43
34
26
15
X
X
THP1 Parent
THP1 NLRP1
THP1 NLRP1 sh-Ctrl
THP1 NLRP1 sh-STING
THP1 Parent sh-Ctrl
THP1 Parent sh-STING

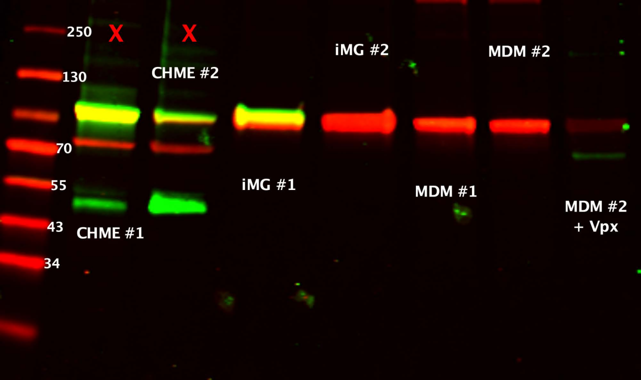
250
130
70
55
43
34
X
X
CHME #1
CHME #2
iMG #1
iMG #2
MDM #1
MDM #2
MDM #2
+ Vpx

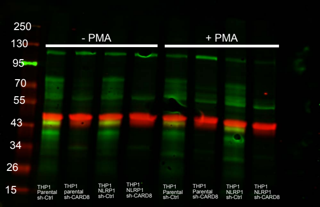
250
130
95
70
55
43
34
26
15
- PMA
+ PMA
THP1 Parental sh-Ctrl
THP1 parental sh-CARD8
THP1 NLRP1 sh-Ctrl
THP1 NLRP1 sh-CARD8
THP1 Parental sh-Ctrl
THP1 Parental sh-CARD8
THP1 NLRP1 sh-Ctrl
THP1 NLRP1 sh-CARD8

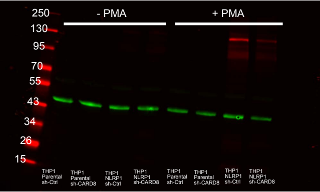

250
130
95
70
55
43
34
26
15
- PMA
+ PMA
THP1 Parental sh-Ctrl
THP1 Parental sh-CARD8
THP1 NLRP1 sh-Ctrl
THP1 NLRP1 sh-CARD8
THP1 Parental sh-Ctrl
THP1 Parental sh-CARD8
THP1 NLRP1 sh-Ctrl
THP1 NLRP1 sh-CARD8
