# Supplementary material for: Expression of intron-containing HIV-1 RNA induces NLRP1 inflammasome activation in myeloid cells
Source: PLoS Biol. 2025 Sep 8;23(9):e3003320. doi: 10.1371/journal.pbio.3003320 (PMC12416851; doi:10.1371/journal.pbio.3003320)
Supplement: S3 Data — (ZIP) [file pbio.3003320.s011.zip › S3_Data/S3_Data_B_iMGs_Characterization_FCS-files/Fig3_Bii_iMGs_P2RY12_FCS-files/Fig3_Bii_iMGs_P2RY12-staining_gating.pdf]

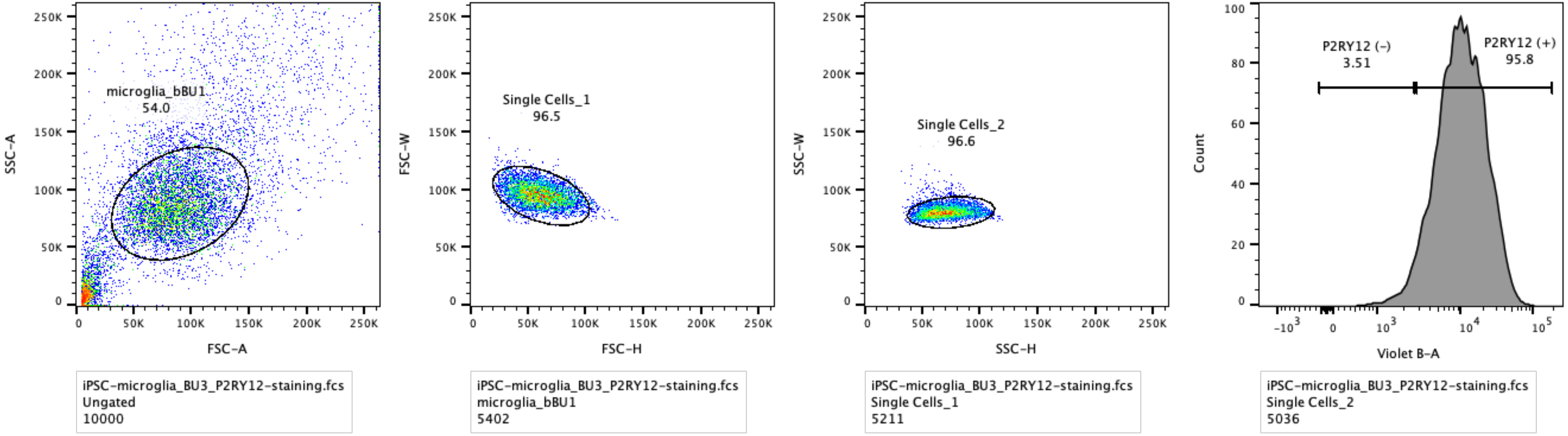

250K
200K
150K
100K
50K
0
SSC-A
FSC-A
0
50K
100K
150K
200K
250K
microglia_bBU1
54.0
iPSC-microglia_BU3_P2RY12-staining.fcs
Ungated
10000
FSC-W
FSC-H
Single Cells_1
96.5
iPSC-microglia_BU3_P2RY12-staining.fcs
microglia_bBU1
5402
SSC-W
SSC-H
Single Cells_2
96.6
iPSC-microglia_BU3_P2RY12-staining.fcs
Single Cells_1
5211
Count
100
80
60
40
20
0
Violet B-A
-10^3
0
10^3
10^4
10^5
P2RY12 (-)
3.51
P2RY12 (+)
95.8
iPSC-microglia_BU3_P2RY12-staining.fcs
Single Cells_2
5036
