# Supplementary material for: Expression of intron-containing HIV-1 RNA induces NLRP1 inflammasome activation in myeloid cells
Source: PLoS Biol. 2025 Sep 8;23(9):e3003320. doi: 10.1371/journal.pbio.3003320 (PMC12416851; doi:10.1371/journal.pbio.3003320)

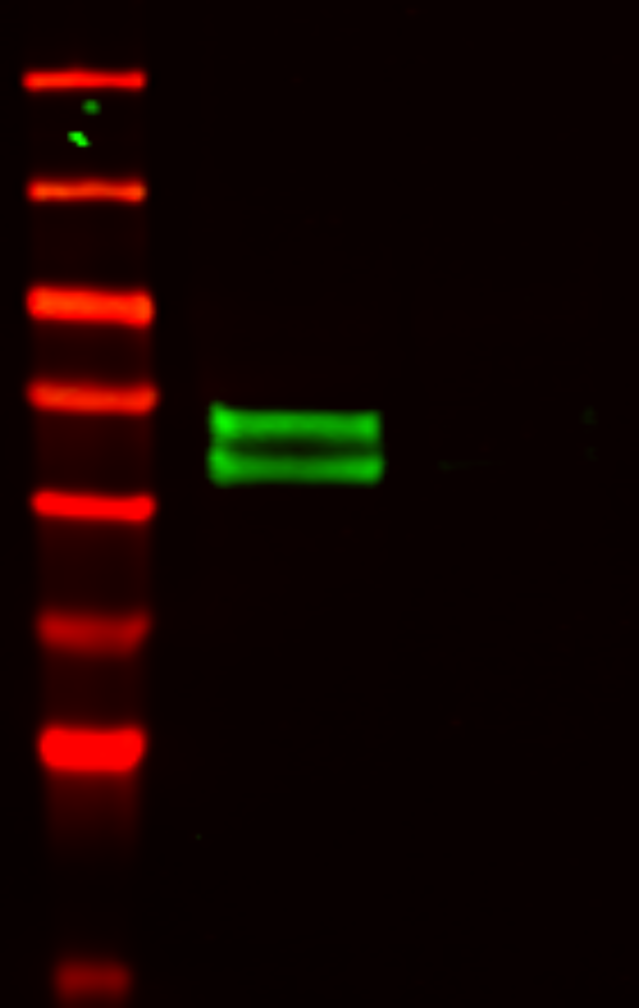

Supplement: S5 Data — (ZIP) [file pbio.3003320.s013.zip › S5_Data/S5_Data_B_CASP1.pdf]
