# Supplementary material for: Expression of intron-containing HIV-1 RNA induces NLRP1 inflammasome activation in myeloid cells
Source: PLoS Biol. 2025 Sep 8;23(9):e3003320. doi: 10.1371/journal.pbio.3003320 (PMC12416851; doi:10.1371/journal.pbio.3003320)
Supplement: S2 Supplementary Data — (ZIP) [file pbio.3003320.s017.zip › S2_Supplementary_data/FigS2_C_iMGs_infections_FCS-files/FigS2_C_iMGs_infections_gating.pdf]

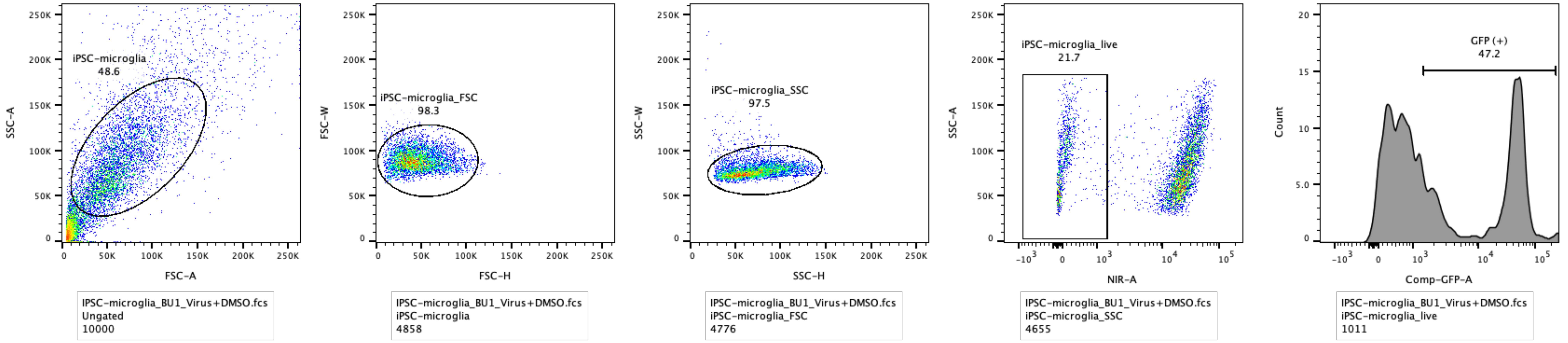
iPSC-microglia
48.6
SSC-A
FSC-A
IPSC-microglia_BU1_Virus+DMSO.fcs
Ungated
10000
iPSC-microglia_FSC
98.3
FSC-W
FSC-H
IPSC-microglia_BU1_Virus+DMSO.fcs
iPSC-microglia
4858
iPSC-microglia_SSC
97.5
SSC-W
SSC-H
IPSC-microglia_BU1_Virus+DMSO.fcs
iPSC-microglia_FSC
4776
iPSC-microglia_live
21.7
SSC-A
NIR-A
IPSC-microglia_BU1_Virus+DMSO.fcs
iPSC-microglia_SSC
4655
GFP (+)
47.2
Count
Comp-GFP-A
IPSC-microglia_BU1_Virus+DMSO.fcs
iPSC-microglia_live
1011
