# Supplementary material for: Expression of intron-containing HIV-1 RNA induces NLRP1 inflammasome activation in myeloid cells
Source: PLoS Biol. 2025 Sep 8;23(9):e3003320. doi: 10.1371/journal.pbio.3003320 (PMC12416851; doi:10.1371/journal.pbio.3003320)
Supplement: Attachment — Submitted filename: Jalloh_etal_Response-Letter_060925.pdf [file pbio.3003320.s020.pdf]

We thank the reviewers for their valuable comments and suggested experiments, which are now included in the revised manuscript. To address the reviewers' concerns, we have included new results which are added in four separate figures, and a schematic to summarize the findings of this manuscript.
Fig. S1A-B: Shows infection efficiency and IL-1β secretion from HIV-1 infected macrophages in the absence or presence of either SIV3+/Vpx-VLPs or dNs.
Fig. 4H and **Fig. 5B:** Western blot analysis to confirm knock-down of inflammasome sensors in siRNA-transfected primary human macrophages.
Fig. S5A-B: Flow cytometry-based caspase-1 activation (FLICA) assay of infected MDM cultures.
Fig. 7C-F: Single molecule RNA FISH and immunofluorescence microscopy to demonstrate cytoplasmic co-localization of unspliced HIV-1 RNA with NLRP1 in macrophages.
Fig. 7I: A basic schematic to describe the diverse HIV-1 RNA-induced inflammatory responses.

Our new results show that HIV-1 icRNA-mediated caspase-1 activation and cell death is observed in both infected cells and bystander cells (**Fig. 5A-B**), and that this activation in virus-infected cells is strictly triggered by cytoplasmic sensing of HIV-1 icRNA by NLRP1. Furthermore, we show that caspase-1 activation is attenuated in the absence of de-novo viral icRNA expression. Using two orthogonal approaches, including RNA-IP followed by qRT-PCR, and now smFISH, we show that HIV-1 icRNA directly co-localizes with cytoplasmic NLRP1 (**Fig. 7C-F**). We include detailed responses to the reviewers' comments below. Reviewers' comments are indicated in blue, our responses in black.

Reviewer #1:

An increasing amount of mechanisms for the detection of HIV RNAs are being reported and may contribute to chronic inflammation observed in PLWH. The authors previously reported that intron-containing HIV RNAs (icRNAs) can be sensed by MDA5/MAVS. Here, they report that in addition, cytosolic icRNAs can be sensed by macrophages and microglia to trigger secretion of IL-1ß. Through a series of conclusive experiments using inhibitors, gene silencing and virus variants, clear evidence is presented that this new mechanism involves NLRP1 and caspase-1 and is distinct from recently described mechanisms involving MDA5/MAVS or CARD8. This is a carefully conducted study that presents interesting and novel findings. The results are clear, for the most part generated using highly relevant primary target cells and the manuscript is very well written. Reflecting this maturity, this seems to be a revised manuscript version. I have not seen the initial submission and will focus on a few general points that could still be improved.

Reviewer #1 Major Comments:

1. A schematic model that puts the newly discovered mechanisms in the context of previous reports would be helpful for the reader.

We appreciate the reviewer’s suggestions, and we have included a basic schematic (**Fig. 7I**) to incorporate our previous reports and present findings on cytosolic HIV-1 icRNA sensing and immune activation in myeloid cells.

2. The efficacy of PRR knockdowns was assessed on the mRNA and functional level. Including an additional analysis on the protein level would be important.

We agree with the reviewer that this would be an important addition. In response to the reviewer’s comment, we evaluated knockdown in siRNA transfected primary macrophages at day 2 post transfection (corresponding to the day of infection) by WB analysis (**Fig. 4H** and **Fig. 5B**). Similar to our initial observations based on RT-qPCR analysis, we observed significant reduction in protein expression of targeted inflammasome components and adaptors.

3. Only little focus is put on the functional consequences of icRNA sensing (LDH release in 5F, 6J). A more detailed analysis of viability of infected cultures and assessing the impact of cell culture supernatants on the susceptibility of naïve cultures to infection would be a good addition.

We thank the reviewer for this suggestion. We utilized the commercially available FLICA assay kit (ImmunoChemistry Technologies, #9122) to fluorescently stain for activated caspase-1 expression in HIV-1-infected THP1/NLRP1-PMA macrophages. As shown in the modified supplementary figure 5 (**Fig. S5A-B**), we observed a marked increase in activated caspase-1 in the infected (GFP +ve) population, which was attenuated upon pre-treatment with reverse transcriptase inhibitor (Efavirenz), indicating death of productively infected cells. While double positive (caspase-1+/GFP+) cells were observed at day 3 pi, most of the GFP+ cells were FLICA negative. Interestingly, a significant number of uninfected cells (GFP -ve) also exhibited caspase-1 activation, which we hypothesize might be induced by transfer of active caspase-1 molecules via exocytic vesicles to bystander uninfected cells, as described in a recent study, as a mechanism of bystander cell pyroptosis (*Wright et al, 2025 Cell 188:280-291; PMID: 39742811*), and propagation of inflammatory responses to uninfected cells. HIV-1 infected MDMs secrete diverse inflammatory mediators, including IL-1β and type I IFNs, amongst others (*Akiyama et al, 2018 Nat Comm 9:3450; PMID: 30150664*). To ascertain the relative contribution of each of these cytokines and chemokines to HIV-1 infection of naïve T cells, while interesting, is beyond the scope of this manuscript.

Reviewer #2:

This is a novel and interesting pathway, and adds a new feature to our understanding of how NLRP1 can trigger inflammasome activation. Broadly, the experiments are well designed and executed, with various inhibitors of key steps of HIV-1 replication being exploited to carefully nail down the pathway driving the outcomes observed. With that

said, I must note that the level of inflammasome activation induced via this pathway appears to be extremely low, especially in MDMs, leading to release of IL-1B levels that, while statistically significant and appear quite reproducible, do not reach the level of induction one sees for many other stimuli in many experiments. Ultimately, I am more interested in understanding how this sensing occurs in the well-controlled and in many cases quite elegant experiments in the study, but I do think it is important to note the modest levels of inflammasome induction observed in these experiments. I leave it to the editor and reviewer to determine how this may impact the outcome of this review.

There are also a few areas of weakness of the manuscript, described below, that should be addressed prior to publication, and a few technical or editorial issues that should also be addressed.

Reviewer #2 Key Issues:

1. In figure S1, and as it related to figure 1, it would certainly be nice to appreciate the level of infection and/or IL-1B induction in cells with neither Vpx or dNs on board. These elements do not seem to be involved in figure 1, but the infection readout (p24) actually comes in higher than with Vpx or dNs treatment. Sine the authors make a point of saying dNs increase levels to the levels seen with Vpx, comparing each to an infection with neither, in the same experiment, seems important here.

We appreciate the reviewer's suggestion here, and we apologize for this oversight in not including all 3 infection conditions (i.e., +Vpx, +dNs, and minus both) in MDMs derived from the same donors. In the absence of either Vpx or dNs, we observed low levels of HIV-1 infection primarily due to SAMHD1 restriction, and lower but detectable levels of IL-1β secretion upon TNFα priming, compared to that observed upon establishment of infection in the presence of Vpx-VLPs or dNs. These results have been added as new panels in supplementary figure 1 (**Fig. S1A-B**), and the manuscript has been updated accordingly.

2. In figure S4, the authors use Immunofluorescence to compare the levels of NLRP1 in THP1s (transduced or not to express NLRP1), MDMs and iPSC derived microglia. Especially given the relatively modest level of response of MDMs, compared to microglia, and other studies which have shown low or undetectable levels of NLRP1 in some of these cell types, I think it is really critical to include a quantitative WB analysis of NLRP1 expression levels in all of these cell types. It appears that perhaps expression is a bit higher in iMGs compared to MDMs, which would ease my concerns related to the modest response in MDMs, as the authors do measure more impressive levels of IL-1β release from iMGs in figure 3.

We agree with the reviewer here. The levels of NLRP1 expression primary macrophages varies depending on the donor, whereas our iMGs were all differentiated from two donors, and they show higher NLRP1 expression compared to MDMs. Given the timeline required to generate iMGs, we believe the immunofluorescence analysis sufficiently shows the differential levels of NLRP1 expression in these distinct cell types,

which underline why untransduced THP1s and MDMs show significantly lower levels of IL-1β secretion compared to iMGs. To address reviewers' concern, we have included western blots of MDM lysates that show NLRP1 expression (**Fig. 5B**), and while the levels are low, we demonstrate that knock-down of NLRP1 specifically in MDMs resulted in abrogation of IL-1β secretion from HIV-infected MDMs (**Fig. 5D**).

3. The mechanistic data in Figure 7 is quite interesting and provocative, and key to understanding how NLRP1 can act as described in the manuscript. While the authors note no colocalization of J2 antibody staining and NLRP1, a proximity ligation assay would be a much more sensitive and potentially informative indicator of association between dsRNA species, presumably parts of the HIV-1 genome with RNA duplexes, and NLRP1.

We agree with the reviewer that this is an interesting observation. The lack of double-stranded RNA co-localization (using J2 antibody) with NLRP1 in our initial studies was undoubtedly due to lack of specificity and sensitivity of the J2 antibody to HIV-1 icRNAs. To address this technical limitation, we utilized a different technology based on single-molecule RNA FISH (smFISH) to label HIV-1 icRNAs directly and specifically in infected cells, in conjunction with immunofluorescence to visualize NLRP1 and HIV-1 $p24^{Gag}$ (as a positive control) expression (now included in **Fig. 7C-F**). This new approach allowed us to detect co-localization of NLRP1 and HIV-1 icRNAs in the cytoplasm and near the plasma membrane, presumably at virion assembly sites. We observed that NLRP1 co-localization with HIV-1 icRNAs was only observed in WT virus-infected cells but not in M10 (Rev mutant) virus infected cells, confirming that it is the de-novo transcribed cytosolic icRNAs (and not the incoming genome) which is interacting with NLRP1.

4. The authors never really address the fate of cells undergoing NLRP1 mediated inflammasome activation, which is a weakness of the manuscript as submitted. Some LDH release data seems to suggest that cells die when this pathway is operative, but this does not seem to influence the number of infected cells in culture (as measured by GFP expression). Are only a very small subset of cells undergoing inflammasome activation via this pathway? Alternatively, are these cells avoiding pyroptotic cell death despite releasing IL-1B. Numerous approaches can be used to better understand this, including ASC spec formation or GSDMD cleavage and localization to the plasma membrane of infected cells. Does activation trigger PI uptake? Understanding the fate of infected cells following NLRP1 mediated inflammasome activation really seems critical to appreciate how this may influence HIV-1 pathogenesis/HAND or HIV-1 associated chronic inflammation, as suggested in the discussion.

As described in the response to reviewer #1's critique, we utilized FLICA assay to measure the extent of caspase-1 activation in infected MDM cultures. As shown in the new data panels in supplementary figure 5 (**Fig S5A-B**), double positive (caspase-1+/GFP+) cells were observed at day 3 pi, though most of the GFP+ cells were FLICA negative. Since EFV pre-treatment reduced the number of FLICA+ cells, undoubtedly, reduction of HIV-1 icRNA expression induced inflammasome activation. Interestingly, a significant number of uninfected cells (GFP -ve) also exhibited caspase-1 activation,

which we hypothesize might be induced by transfer of active caspase-1 molecules to bystander uninfected cells, by exocytic vesicles (*Wright et al, 2025 Cell 188:280-291; PMID: 39742811*), and propagation of inflammatory responses to uninfected cells. It remains formally possible that FLICA assay might not capture all the double positive cells, as dead cells might have been lost during the wash steps of the experimental protocol, thus resulting in lower numbers of caspase-1+/GFP+ double positive cells. Alternatively, as suggested by the reviewer, since both gasdermin-D (GSDMD)-dependent and independent mechanisms have been proposed to facilitate IL-1β secretion, relatively small numbers of the caspase-1+ infected cells could contribute to IL-1β secretion and dissemination of inflammatory responses, Understanding the kinetics and interplay between this inflammasome pathway and other active pathways such as type-I interferons in the context of HIV-1 infection in myeloid cells will ultimately lead to a better understanding of the fate of productively infected cells both in vitro and in vivo.

Reviewer #2 Minor Issues:

5. Figure 1: In panel H, can the channels of the western blot be separated into capase-1 only and actin only panels. The point will not change, but the combination is not particularly aesthetically pleasing. Same comment for 2A.

We appreciate this feedback and have made the suggested changes accordingly.

6. The generation of the delta Gag-Pol virus is rather poorly described. In the materials and methods, it is stated: Single-round viruses pseudotyped with VSV-G were generated from HEK293T cells via co-transfection of LaiΔenvGFP proviral plasmids and VSV-G expression plasmid (pHCMV-G), and the packaging construct (psPAX2), where necessary. It would be worth clarifying which viruses were prepared with a packaging plasmid assist. Additionally, it might be worth a few lines of text in the result clarifying that this virus was made in 293Ts with a packaging plasmid because it is not implausible that a reader without substantial expertise in HIV-1 molecular and cellular approaches would be very confused about how a delta Gag/Pol virus could be made or be infectious.

We apologize for this oversight. The generation of the different mutant viruses including ΔGag-Pol is now described in the methods section (**lines 181-182, lines 204-208,** and **lines 600 – 613**). Just for clarification, we co-transfected HIV-1 Gag-Pol helper packaging plasmid, psPAX2, with proviral plasmids that either expressed inactive Rev (M10) or expressed Gag-pol deletion (ΔGag-pol) to rescue infectious virus production from HEK293T cells. These viruses which were used for MDM infections successfully established infections, but do not express Gag or Gag-Pol polyproteins in target cells.

7. Line 76, note oddly formatted citations. Likely a formatting error, but if not, Im not clear on what this would mean.

We apologize for this oversight and have now fixed the formatting error.

8. Line 369: Figure 4 is called out, should be figure 5.

We apologize for this oversight and have now fixed the figure error.

Reviewer #3 (Jeremy Luban):

The literature has many reports of chronic inflammation, including inflammasome activation, in people living with HIV-1. Previously, the authors showed that unspliced, intron-containing HIV-1 RNA (icRNA) activates type 1 IFN in myeloid cells, including monocyte-derived macrophages (MDMs) and iPSC-derived microglia (iMGs). Here, they show that HIV-1 icRNA activates the inflammasome in these cells, as evidenced by IL-1B secretion and Caspase 1 cleavage. HIV-1 protease activity was not required for inflammasome activation. siRNAs targeting MAVS, STING, and UNC93B1 had no effect on HIV-1 icRNA-induced IL1B secretion. Previous work has shown that Rev-RRE-CRM1 is required for type 1 IFN induction by HIV-1 icRNA and that export of icRNA via a 4x-CTE would not substitute; here they show that the 4X-CTE is sufficient to activate IL1B. Knockdown of NLRP1 and CASPASE1, but not AIM2, NLRP3, CARD8, or caspase-4, decreased HIV-1 icRNA-induced IL-1β secretion and LDH release. THP1 cells do not produce NLRP1 protein and do not make IL1B in response to HIV-1 icRNA; when THP1 cells were transduced with an NLRP1-expressing lentivector, they did. This manuscript reports an important discovery: HIV-1 icRNA is detected by NLRP1 in myeloid cells and activates the inflammasome to secrete IL1B. The experiments are well controlled, the data convincing, and the manuscript is clear. Our suggestions are minor and can be quickly remedied.

Reviewer #3 (Jeremy Luban) Major Comments:

1. Page 4, line 81 mentions MDA5 without the relevant references. Refs 85 and 86 should be added here.

We appreciate this feedback and have added the relevant references as suggested.

2. Page 5, line 103 suggests that IP10 data is shown in Fig 1. The authors could just indicate that this was previously shown (ref 56).

We acknowledge this lack of clarity. We have re-written the sentences to clarify that the IP-10 data is not shown, we just referenced our previous findings.

3. Fig 4F-J might be better split off as a separate figure that comes before Fig 4A-E. Panels 4F-J are about host factors and would flow better into Fig 5, which is also about host factors.

This is a good suggestion. We have now re-ordered the panels in this figure, as suggested by the reviewer. Figure 4 now flows better into figure 5.

4. It is a little hard to distinguish the identities of the different bars in Fig 5B-F based on the color code. Perhaps the bars could be labeled as they were in panel A

We appreciate this feedback and have made the changes on the symbols in the respective bars to clarify the differences.

5. Figure 6: All figures prior to this one show statistical significance. Is IL1B induction by HIV-1 icRNA significantly above background?

We acknowledge the Reviewer's suggestion and have added the relevant statistical analysis for these figures. And yes, we observed that overexpressing NLRP1 in THP1 monocytes via lentiviral transduction increased the baseline background level of IL-1β secretion in mock (uninfected) cells, though we note that the elevated background level of IL-1β in this cell line does not change our conclusions.

6. Page 21, line 491: the authors state that dsRNA and NLRP1 colocalization was not detected. Would it be possible to detect colocalization using a proximity ligation assay? Such an experiment is not necessary but it would be fun to see the results.

As already discussed in response to Reviewer #1's comments, we employed a more robust viral RNA staining technique (smFISH) and observed distinct co-localization of HIV-1 usRNA (icRNA) with NLRP1 (**Fig. 7C-D)**).
